# The three-dimensional structure of Epstein-Barr virus genome varies by latency type and is regulated by PARP1 enzymatic activity

**DOI:** 10.1038/s41467-021-27894-1

**Published:** 2022-01-17

**Authors:** Sarah M. Morgan, Hideki Tanizawa, Lisa Beatrice Caruso, Michael Hulse, Andrew Kossenkov, Jozef Madzo, Kelsey Keith, Yinfei Tan, Sarah Boyle, Paul M. Lieberman, Italo Tempera

**Affiliations:** 1 The Wistar Institute, Philadelphia, PA USA; 2 Fels Institute for Cancer Research and Molecular Biology, Lewis Katz School of Medicine at Temple University, Philadelphia, PA USA; 3 University of Oregon, Eugene, OR USA; 4 The Coriell Institute for Medical Research, Camden, NJ USA; 5 Fox Chase Cancer Center, Philadelphia, PA USA

**Keywords:** Molecular biology, Virology

## Abstract

Epstein-Barr virus (EBV) persists in human B-cells by maintaining its chromatinized episomes within the nucleus. We have previously shown that cellular factor Poly [ADP-ribose] polymerase 1 (PARP1) binds the EBV genome, stabilizes CTCF binding at specific loci, and that PARP1 enzymatic activity correlates with maintaining a transcriptionally active latency program. To better understand PARP1’s role in regulating EBV latency, here we functionally characterize the effect of PARP enzymatic inhibition on episomal structure through in situ HiC mapping, generating a complete 3D structure of the EBV genome. We also map intragenomic contact changes after PARP inhibition to global binding of chromatin looping factors CTCF and cohesin across the EBV genome. We find that PARP inhibition leads to fewer total unique intragenomic interactions within the EBV episome, yet new chromatin loops distinct from the untreated episome are also formed. This study also illustrates that PARP inhibition alters gene expression at the regions where chromatin looping is most effected. We observe that PARP1 inhibition does not alter cohesin binding sites but does increase its frequency of binding at those sites. Taken together, these findings demonstrate that PARP has an essential role in regulating global EBV chromatin structure and latent gene expression.

## Abstract (web summary)

Genome folding in the nucleus plays an important role in regulating gene expression. Here the authors show that 3D genome architecture also plays an important role for gene expression adaptability for the Epstein-Barr Virus (EBV), a virus that is associated with cancer. They also observe major changes in the fold of the EBV chromosome between silent and transcriptional active viral genomes.

## Introduction

Epstein-Barr virus establishes lifelong latency in human B-cells through maintenance of its chromatinized episomes replicating in synchrony with the host genome via cellular and viral factors^1^. These circularized mini-chromosomes do not integrate into the host genome, making it necessary for EBV to organize its chromatin in a manner that allows access to genes essential for transcription and replication, yet structured enough to maintain genomic stability. As EBV has at least four established latency-associated transcriptional repertoires, the maintenance of a stable 3D chromatin structure for each program may explain the stability of latency-specific gene expression. In healthy adults, EBV exists in its proper latent form, “latency 0”, or the minimally transcriptionally active form “latency I” within resting memory B-cells^1^. During latency 0, only noncoding RNA is transcribed from the episome, including BART, BHRF1, and EBER microRNAs^1^. Type I latency only differs in the expression of one viral protein, EBV nuclear antigen protein 1 (EBNA1), which acts as a transcription factor to replicate the episome during host cell division^1^. There are numerous malignancies associated with type I EBV latency, which typically arises in immunocompetent individuals, including Burkitt’s lymphoma and gastric carcinoma^2^. An intermediate transcriptional profile, “latency II”, expressing EBNA1 and the noncoding RNAs in addition to latent membrane proteins (LMP1, 2a, and 2b) has been occasionally reported within the germinal center of healthy individuals. It is also associated with several malignancies, including nasopharyngeal carcinoma, Hodgkin’s disease, and nasal T/natural killer-cell lymphomas^1–3^. The final and most transcriptionally active latency program, “latency III”, expresses all previously mentioned genes in addition to various other latency transcripts^1^. Because 1–1.5% of all human cancers are associated with EBV infection^4–6^, there is an apparent need for EBV-specific treatments of these diseases. The current standard of care does not account for potential viral driving factors or etiology^7^. As each EBV latency program has a specific subset of associated malignancies, understanding how EBV maintains latency type may provide opportunities for novel, targeted therapeutics.

The host enzyme poly [ADP-ribose] polymerase 1 (PARP1) catalyzes the addition of ADP-ribose polymers of varying lengths to histone H1 as well as various target proteins^8^. Our group and others have previously shown that PARP1 and PARylation have an established role in latency type maintenance and lytic reactivation of EBV via regulation of the chromatin-binding protein CTCF^9–12^. We have also recently published that PARP1 colocalizes with CTCF across the EBV episome and that PARP1 enzymatic activity stabilizes CTCF binding and maintains a permissive chromatin status at the C-promoter (Cp) during type III latency^10^.

CTCF is a cellular factor that regulates chromatin composition and the three-dimensional structure of the genome by promoting chromatin loop formations^13^. CTCF functions as both an insulator to prevent the spread of epigenetic marks between various chromatin loops and as an anchor to recruit cohesin, which physically forms chromatin loops via its ring-shaped SMC components holding two separate regions of chromatin together^14^. In the human genome, CTCF insulator function is known to be enhanced by PARP1 at specific loci, and CTCF, in turn, activates PARP1 enzymatic activity^15–17^. Previous work has established a role between both CTCF and cohesin^11,18–21^, and CTCF and PARP1^9,10^ in regulating the epigenome of EBV. Because of these findings, in the present work, we aimed to investigate whether inhibition of PARP1 catalytic activity would disrupt the global EBV genomic structure, specifically by altering the binding of the chromatin looping factors CTCF and cohesin. As cohesin is necessary for CTCF to form long-range intragenomic interactions^22–24^, and there is evidence that CTCF colocalizes with cohesin across the EBV genome^19^, we postulated that PARylation may not only regulate the binding of CTCF to the EBV genome, but also cohesin, and perhaps alter the interaction between these two proteins to influence gene expression^14,18^. Here, we report that PARP inhibition surprisingly stabilizes the binding of cohesin at CTCF/cohesin colocalization sites. We also found that isogenic type I and III EBV genomes have nearly identical CTCF and cohesin binding profiles. Despite this, the 3D conformation of the type I and type III genomes was strikingly different, with only seven DNA-DNA interactions in type I and 26 in type III. Inhibition of PARylation alters the EBV episome’s 3D conformation, with long-range chromatin looping lost in the type I genome and a distinct intragenomic binding profile observed in the type III genome. When PARylation is inhibited in type III latency, we witnessed the downregulation of viral genes typical of the type III latency repertoire and an increased expression of lytic genes. The data presented in this study illustrate a role for PARP1 in the regulation of 3D chromatin structures and cohesin-chromatin binding, as well as maintenance of EBV latency type.

To date, the effect of PARylation on cohesin-chromatin binding has yet to be studied in the context of EBV or the human genome. Nor has the effect of PARP inhibition on 3D chromatin remodeling, i.e., chromatin looping, been studied on a genome-wide level. Thus, our work investigating the impact of PARP inhibition in EBV-positive B-cells is of high significance not only for its therapeutic potential in the context of host/viral interaction but also for providing a better understanding of host chromatin modifiers on a small scale, offering higher resolution than is currently possible in the human genome.

## Results

### 3D chromatin conformation varies by latency type in isogenic EBV genomes

**Fig. 1:**
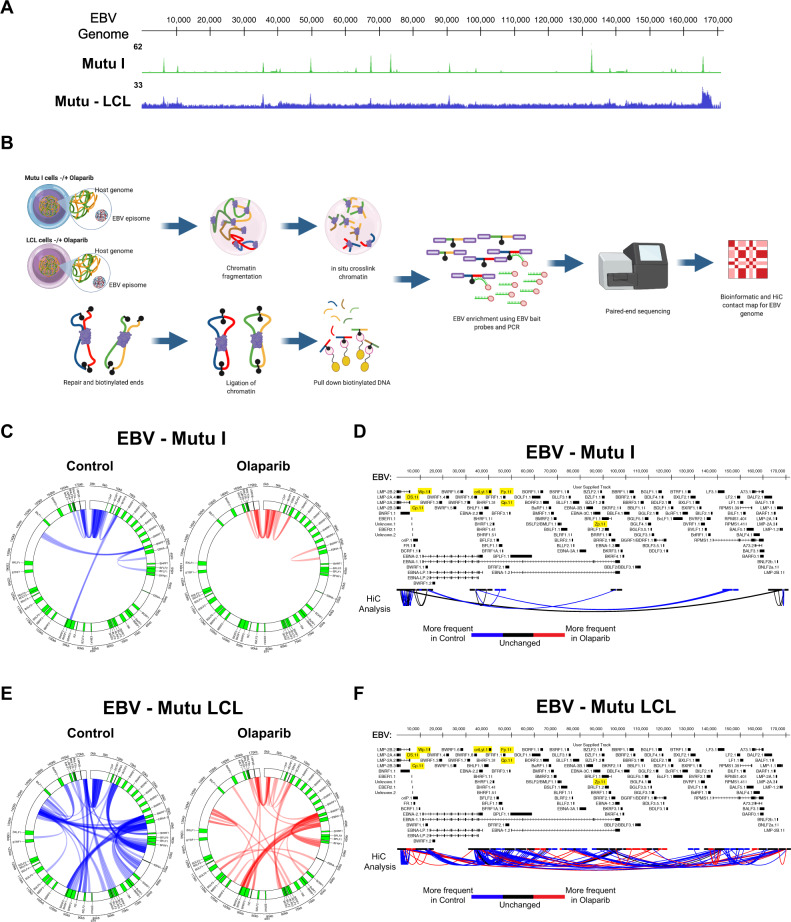
EBV 3D chromatin conformation is altered by latency type and PARP inhibition. **A** CTCF ChIP-seq profiles in human EBV+ B-cells with type I latency (Mutu, green) or type III latency (LCL, blue), normalized to input chromatin. **B** Schematic of HiC workflow followed by EBV enrichment and sequencing. The figure was created with BioRender. **C** Circos graphs of all DNA-DNA contacts with the significance of <0.05 within the type I latency EBV genome (Mutu) derived from HiC matrices. The left circos graph represents the control (untreated genome, blue) and the right represents the type I genome with 2.5 µM olaparib treatment (red). **D** Linearized type I EBV genome (Mutu) with arcs connecting DNA-DNA contacts derived from HiC matrices. Blue arcs represent chromatin loops that are more frequent in the control genome, red arcs represent chromatin loops that are more frequently observed in the olaparib treated genome, and black arcs are loops that are unchanged between control and treatment. **E** Circos graph (as described in (**C**)) for untreated (left, blue) and olaparib treated (right, red) type III EBV genome (LCL). **F** Linearized type III EBV genome (LCL) as described in (**D**).

We and others have demonstrated that during type III EBV latency, CTCF binds the EBV genome at several regions and regulates viral gene expression^9–11,19,20,25^. Since changes in viral gene expression are associated with different EBV latency programs, we investigated whether differences in CTCF occupancy across the viral genome exist between different latency types. To compare CTCF binding across the EBV genome in type I and type III latency programs, we performed chromatin-immunoprecipitation and global sequencing (ChIP-seq) on a human type I latency B-cell line (Mutu I) and compared it with a type III profile we previously generated that is isogenic with respect to the EBV genome^10^. When the type I CTCF binding profile was aligned to the published type III profile, we observed minimal differences (Fig. 1A), except for the ~132 kb peak observed in the type I Mutu genome that is absent in the type III Mutu-LCL. These data indicate that differences in CTCF binding are unlikely to contribute to the differences in viral gene expression observed between EBV latency types. CTCF regulates gene expression by promoting chromatin loop formation in mammalian genomes^26^. Previous studies have demonstrated that CTCF occupancy promotes chromatin loop formation between specific regions of the viral genome^25,27^. To extend the analysis of chromatin loops existing between other CTCF-associated regions and to explore the hypothesis that the three-dimensional structure of the viral genome differs between different latency types, we assessed total EBV genomic structure by in situ HiC assay^28^, followed by enrichment for EBV–EBV chromatin interactions (Fig. 1B). Sequenced reads (ligation productions of DNA–DNA physical interactions) were aligned to the EBV genome using Bowtie2 with iterative alignment strategy and plotted as circos graphs. The darkness of the blue arcs (Fig. 1C, E) represents the strength/frequency of the interaction, with darker loops representing the higher frequency of contact between the two regions the arc connects. Considering CTCF’s established role as a chromatin looping factor, we were surprised to observe significantly fewer chromatin loops in type I latency compared to type III despite the very similar ChIP-seq tracks for CTCF (Fig. 1C–E). When looking for similarities between the latency I and III genomes, only two chromatin interactions remain entirely conserved: 1) the dense local interactions between the terminal repeat, TR, region (~170 kb), and the region directly upstream of the origin of viral replication (~2.5 kb) and 2) the 10 kb loop formed between the lytic transcripts BWRF1 (~35 kb) and BFLF1 (~46 kb) (Fig. 1C, E). The two long-range chromatin loops formed between BCRF2 (~13 kb) and BKRF1 (~96.5 kb), and between BWRF1 (~35 kb) and the BART microRNAs (~143 kb) in the type I latency genome has been completely lost in the type III genome. However, many loops and local interactions are formed in type III latency that is not observed in the type I genome. The strongest interactions in type III latency localize around the C-, Z-, and LMP- promoters, within the EBNA transcripts, and surprisingly within the BART microRNAs (Fig. 1E, F). Overall, we observed seven total unique interactions in the type I episome and 26 in type III (Supplementary Data 1), accounting for the fact that numerous overlapping and nearly overlapping interactions visible on the circos graphs may be the same event captured multiple times. On a technical note, we found the two biological replicates of the HiC assays to be highly similar when comparing the raw matrices, providing confidence in the inferred episomal structure (Supplementary Data 2). Altogether, these results indicate that the three-dimensional organization of the EBV genome differs significantly between isogenic type I and type III latency types, with more chromatin loops existing in type III than type I.

### Intragenomic interactions are globally altered by PARP1 enzymatic inhibition

Our lab has previously shown that the more transcriptionally active type III latency has three to fourfold higher intracellular PAR (pg/mL) than the immunoevasive type I latency^12^. As PARP1 has an established role in chromatin remodeling, primarily by regulation of CTCF insulator function^16^, we investigated whether the reported increase in PARP activity was necessary for the maintenance of EBV episomal 3D structure. We additionally wanted to assess if the higher PARP1 activity in type III latency will confer a higher dependence on PARylation to maintain the highly organized chromatin structure we observed by HiC. To determine the impact of PARP1 activity on EBV chromatin structure, we inhibited PARP enzymatic activity in the same EBV+ B cell lines that are isogenic with respect to the EBV genome. Both cell lines were treated with 2.5 µM of the PARP inhibitor olaparib^29^ and assessed for PARylation levels after 72 h via ELISA. At this concentration, Mutu I PAR levels was decreased 36.06% and LCL PAR levels were decreased 86.24% (Supplementary Fig. 2A). As previously published, the higher PARylation levels in type III latency do not correlate to higher PARP1 protein levels as there is no discernable difference in Mutu I and Mutu-LCL immunoblots for PARP1 (Supplementary Fig. 2B). PARP1 protein levels were also not significantly altered by olaparib treatment, as expected by olaparib’s function as a competitive inhibitor of NAD+, the essential cofactor for PARP1 catalytic activity (Fig. 5C). To determine the effect of the observed reduction in PAR levels and therefore in PARP1 activity, we assessed changes in the 3D structure of the EBV genome by HiC assay as described above. For both type I and type III latency, total unique intragenomic interactions (chromatin loops) with at least a significance of *p* < 0.05 decreased from 7 to 4 (Fig. 1C, D) and from 26 to 18 (Fig. 1E, F, Supplementary Fig. 1 1.368596), respectively, after PARP inhibition. In type I latency, long-range chromatin loops between the ~35 kb region and the 145–150 kb BART microRNA region, and ~13 kb region and the 95–100 kb region disappear, indicating that these regions are no longer interacting (Fig. 1C, D). We also observed a relatively smaller loop disappear between the 35 kb region and the 45 kb region. Interestingly, we observed that no new interactions are gained after olaparib treatment in type I EBV+ cells (Fig. 1C, D). For type III latency, while eight unique high-frequency loops disappear, there are also many regions where new long-range chromatin loops are formed between regions that are on average 60 kb apart (Fig. 1, Supplementary Fig. 1 and Supplementary Fig. 3). In addition, the local interactions within the 142–152 kb region seem to be shifted after PARP inhibition, with a dense region of local interactions now observed from 137 to 143 kb. While the conformation of the type III genome is significantly altered after olaparib treatment, the strong, sequential interactions between the region within the EBNA1.1 transcript directly preceding OriLyt (~33–37 kb) and the region between the lytic Zp and EBNA1.3 (~93–95 kb) remain unchanged, indicating that this chromatin interaction occurs independently of PARP activity. However, when an additional statistical parameter of FDR ≤ 0.05 (Supplementary Data 1) is added atop the *p* < 0.05 threshold (Fig. 1), we see that the type III genome maintains the same absolute number of intragenomic contacts. The seventeen DNA–DNA interactions are not static between the two groups though, as the identity of the fragments is distinct between treatment and control. This statistical discrepancy could indicate that the nine loops not meeting the FDR threshold could be weaker interactions or that these loops are present in only a portion of the five-million cell population. Regardless, our analysis demonstrates that PARP inhibition changes the three-dimensional structure of the EBV genome, particularly in type III latently infected cells.

### Transcription of viral proteins is altered after PARP inhibition

**Fig. 2:**
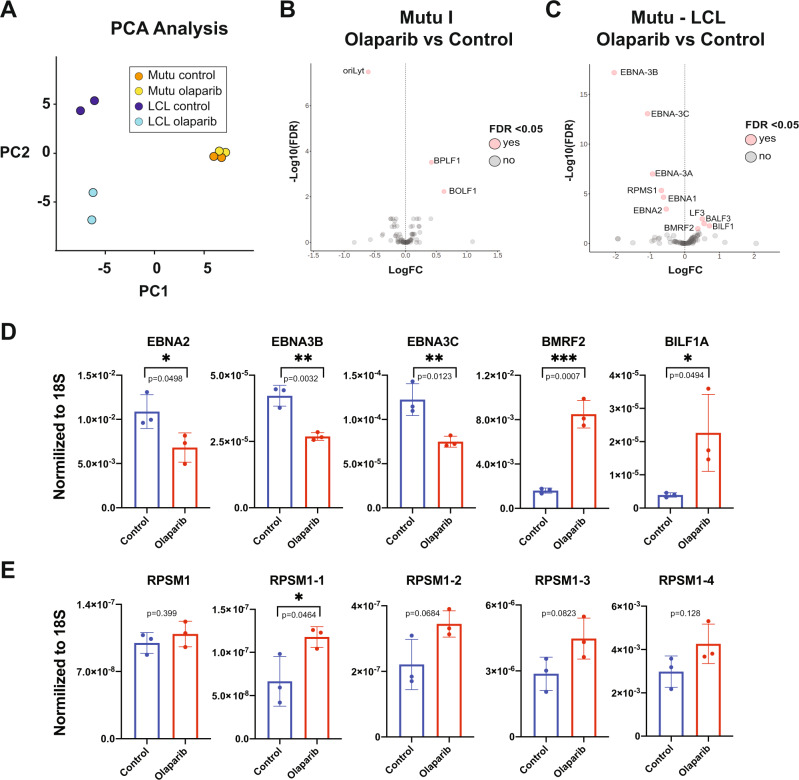
Viral gene expression is dysregulated after PARP inhibition. **A** Principal component analysis (PCA) separating type I EBV latency (Mutu) and type III EBV latency (LCL) human B-cells by treatment (control or 2.5 µM olaparib). Data derived from RNA-seq dataset utilizing biological duplicates. **B** Volcano plot illustrating dysregulated gene expression after olaparib treatment in Mutu cell line, with the left of the dashed line representing genes downregulated after PARP inhibition and the right representing genes upregulated after PARP inhibition. Genes displayed are dysregulated at *p* < 0.05 and FDR < 0.05. Genes with 2-fold change and *q* < 0.01 after correction for multiple testing (FDR) were considered as significantly differentially expressed. **C** Volcano plot illustrating dysregulated gene expression after olaparib treatment in LCL, as described in (**B**). Genes with 2-fold change and *q* < 0.01 after correction for multiple testing (FDR) were considered as significantly differentially expressed. **D** RT-qPCR validation of genes shown to be dysregulated by RNA-seq in LCL after PARP inhibition. Bar graph represents the average expression of three biological duplicates per treatment, each normalized to 18S expression, respectively. (*N* = 3, mean ± SD). Groups were compared by paired student’s *t* test assuming equal variance (two-tailed). (*=*p* ≤ 0.05, **=*p* ≤ 0.01, ***p =≤0.001) Source data are provided as a Source Data file. **E** RT-qPCR validation of RPMS1 expression using five separate primer sets, spanning various exons in the gene. Source data are provided as a Source Data file. The bar graph represents the average expression of three biological replicates per treatment (*N* = 3, mean ± SD). Groups were compared by paired student’s *t* test assuming equal variance (two-tailed). (*=*p* ≤ 0.05, **=*p* ≤ 0.01, ***p =≤0.001).

To determine if the differences in EBV chromatin structure observed after olaparib treatment functionally correlate to altered gene expression, we analyzed changes in viral transcription by RNA-sequencing on biological duplicates of the same type I and III cells used for our HiC assays, with and without PARP inhibition (Fig. 2). The changes in viral gene expression are summarized by principal component analysis (PCA) and a volcano plot (Fig. 2A–C). Principal component 1 separates samples by latency type, with the type III Mutu-LCL samples and the type I Mutu samples diverging along the X-axis (Fig. 2A). Principal component 2 separates LCL samples by treatment but not Mutu samples, which instead grouped together regardless of treatment (Fig. 2A). This is unsurprising considering olaparib treatment in type I cells only reduced intracellular PAR levels 36% compared to type III which was reduced 86% at the same concentration (Supplementary Fig. 2). We next applied a stringent filtering approach (adjusted *p* value < 0.05) to compare changes in the expression of viral genes in EBV+ cells before and after olaparib treatment. In type I Mutu cells, which basally have a minimal transcriptional profile, inhibition of PARP only elicits the differential expression of three genes, including the two lytic genes *BOLF1* and *BPLF1*, which code for the inner and larger tegument protein of EBV respectively^30^ (Fig. 2B). When we applied the same parameters to type III cells, we identified ten viral genes with significantly altered expression after olaparib treatment (Fig. 2C). Specifically, we observed downregulated expression for all EBNA genes, including EBNA2, which we previously reported to be regulated by PARP1^10^. Olaparib treatment also triggers over-expression of four lytic genes, including BALF3 which codes for the viral translocase^31^, BILF1 which codes for the viral G protein-coupled receptor involved in modulating host immunity^32–34^, and BMRF2 encoding the viral protein responsible for EBV attachment to epithelial cells^35,36^. We also observed downregulation of the RPMS1 transcript, from which the BART microRNAs are spliced (Fig. 2C). We next validated the altered expression of the viral genes identified in LCL via RT-qPCR (Fig. 2D, E). Interestingly, four of five separate primer sets used to validate the under-expression of RPMS1 showed no significant difference in expression, while one showed over-expression in olaparib treatment compared to control (Fig. 2E). Because 22 pre-miRNAs are spliced from the RPMS1 transcript, and the various primer sets spanned different exons throughout the gene, we wondered if the regions we chose happened to coincide with differentially expressed microRNAs. Seven miRNAs were selected randomly and compared via RT-qPCR with highly specific locked nucleic acid primers (Supplementary Fig. 4). We found that two of the miRNAs were downregulated at values just above significance at *p* < 0.05, four were unaffected, and one was significantly upregulated. These data are particularly interesting as PARP1 has recently been implicated in alternative splicing^37^. Overall, our results demonstrate that the type III latency gene expression program of EBV is sensitive to PARP inhibition and that PARP activity supports the expression of latent genes while repressing some lytic genes.

### Cohesin–chromatin binding is stabilized by PARP inhibition

**Fig. 3:**
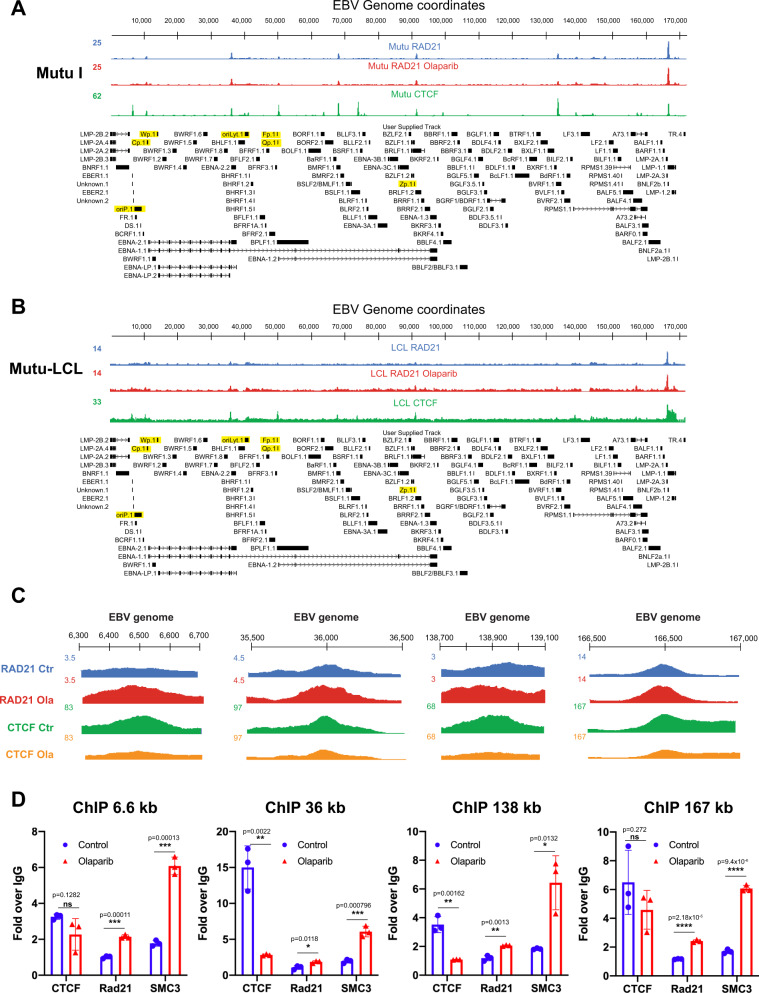
Cohesin–chromatin binding frequency is increased after PARP inhibition. **A** ChIP-seq tracks for CTCF (green), RAD21 (cohesin component, blue), and RAD21 after PARP inhibition (cohesin component, red) in type I EBV latency cell line Mutu. All ChIP-seq tracks are normalized to input chromatin and aligned to the EBV genome. **B** ChIP-seq tracks (as described in (**A**)) in type III latency EBV cell line LCL. **C** Magnified RAD21 binding profiles in LCL, before (blue) or after PARP inhibition (red), aligned to CTCF binding profiles before (green) or after PARP inhibition (orange) in the same cell line. **D** ChIP-qPCR validation of magnified regions of LCL ChIP-seq tracks for cohesin (RAD21, SMC3) and CTCF, normalized to input chromatin and fold change over IgG. Source data are provided as a Source Data file. Values are averaged from three independent assays (*N* = 3, mean ± SD). Groups were compared by paired student’s *t* test assuming equal variance (two-tailed). (*=*p* ≤ 0.05, **=*p* ≤ 0.01, ***=*p* ≤ 0.001).

Our transcriptome analysis revealed the global effect of PARP inhibition on EBV gene expression and confirmed our previous observation about the role of PARP on regulating EBNA2 expression^10^. Previous work also demonstrated that PARP activity is necessary to stabilize CTCF binding across the EBV genome^10^, suggesting that some transcriptional changes we observed could be due to change in CTCF binding and CTCF-mediated chromatin loops. The cohesin complex is known to be anchored by CTCF to promote long-range chromatin loops^28,38^. As cohesin has been shown to colocalize with CTCF across the type III EBV genome^18,19^, we wanted to determine if cohesin binding on the viral genome is also altered by PARP inhibition. To assess cohesin binding before and after PARP inhibition, we treated the same type I and type III cell lines infected with isogenic EBV strains described previously with olaparib. After 72 h, we performed chromatin-immunoprecipitation followed by next-generation sequencing (ChIP-seq) using antibodies against the RAD21 component of the cohesin complex^39^. We normalized the RAD21 binding profile to input chromatin and used peak calling to illustrate cohesion occupancy across the EBV genome in both type I and type III cells lines (Fig. 3). When we analyzed the RAD21 binding profile control cells, we observed that in both types of latency, cohesin colocalizes with CTCF. We observed only one region around 75 kb where CTCF binds alone in both latency types (Fig. 3A, B). Next, we assessed the effect of PARP inhibition on RAD21 occupancy by comparing the binding profile between control and olaparib-treated cells. In the type I Mutu cell line, we observed no significant differences in RAD21 occupancy between treatment groups (Fig. 3A). In type III Mutu-LCL cells, we found that RAD21 occupancy was increased after olaparib treatment at nearly all binding sites across the type III EBV genome (Fig. 3B and Supplementary Fig. 5). Since we previously reported that CTCF binding was reduced at specific regions across the type III EBV genome, we also overlaid our RAD21 ChIP-seq binding profile with previously generated CTCF binding profiles^10^ in the same type III cell line, with and without PARP inhibition (Fig. 3C). We noted that, after PARP inhibition, RAD21 binding is increased at sites where CTCF binding is decreased (Fig. 3C and Supplementary Fig. 5). We validated increased occupancy of cohesin complex at these regions by quantitative ChIP analysis utilizing RAD21 and SMC3 antibodies, and additionally validated CTCF binding at the same regions (Fig. 3D). We observed an increase in occupancy of both cohesion subunits at these viral regions after PARP inhibition as well as a significant decrease in CTCF binding. Next, we pharmacologically inhibited poly(ADP-ribose) glycohydrolase (PARG), the enzyme responsible for PAR moiety breakdown with the specific inhibitor PDD00017273^40^ (Supplementary Fig. 6A). Though this treatment is not the complete opposite to olaparib as PDD does not increase PARP1 activity but rather inhibits the activity of its opposing enzyme, this allowed us to determine if stabilization of PAR chains is enough to decrease cohesin binding frequency or increase CTCF binding frequency. At the previously assessed regions where CTCF binding decreases and cohesin binding increases after PARP inhibition, we now see that cohesin binding is significantly decreased with PDD treatment (Supplementary Fig. 6C). Surprisingly, CTCF binding was not significantly changed by PAR moiety stabilization, indicating that PARylation has an indirect effect on its binding, or that perhaps PARP1 physical binding is more important than its catalytic activity, as has been previously published^16^ (Supplementary Fig. 6B).

Others have reported that olaparib treatment traps PARP on DNA, therefore we assessed the “PARP-trapping” effect of olaparib^41^ at eight sites where CTCF/cohesin colocalize with PARP1 on the EBV genome by quantitative ChIP. We observed increased PARP1 binding frequency at each site assessed after olaparib treatment, though not at a level of statistical significance, indicating that increased physical presence of PARP1 at these sites is unlikely to contribute to the stabilization of cohesin binding (Supplementary Fig. 7). Overall, our results demonstrate that cohesin and CTCF colocalize across the EBV genome in type I as well as type III latency, and that in type III latency cohesin occupancy is regulated by PARP1 enzymatic activity. Our results also revealed that after PARP inhibition, cohesin occupancy increases at regions where CTCF occupancy is reduced.

### Dysregulated viral gene expression is directly correlated with altered EBV chromatin looping

**Fig. 4:**
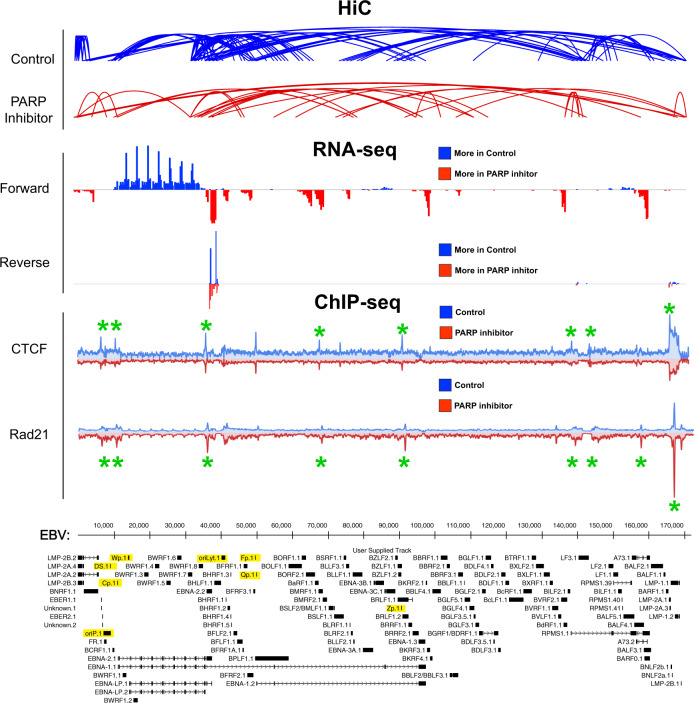
HiC, ChIP-seq, and RNA-seq aligned datasets. The top panel “HiC analysis” aligns differences in chromatin looping between control and 2.5 µM olaparib treated LCLs to the EBV genome. Blue arcs represent chromatin loops that are more frequent in the control genome, red arcs represent chromatin loops that are more frequently observed in the olaparib treated genome, and black arcs are loops that are unchanged between control and treatment. All arcs represent DNA-DNA interactions that occur at a statical significance of *p* < 0.05 from averaged biological duplicates. The middle panel “RNA-seq” displays RNA reads, forward and reverse, aligned to the EBV genome. Blue peaks pointing upward represent reads that occur more frequently in control LCLs while Red peaks pointing downwards represent reads occurring more frequently in olaparib treated LCLs. Genes with 2-fold change and *q* < 0.01 after correction for multiple testing (FDR) were considered as significantly differentially expressed. Bottom panel “ChIP-seq” aligns CTCF and cohesin (RAD21) tracks to the EBV genome, with and without PARP inhibition (2.5uM olaparib treatment for 72 h). The blue peaks pointing upwards represent regions of chromatin where CTCF or RAD21 binds more frequently in control samples while the red peaks pointing downwards represent regions where CTCFor RAD21 binds more frequently after PARP inhibition, respectively. Green stars represent peaks that were determined to be statistically significant binding sties via peak-calling. MACS2 software packages were used to call reads enrichment in pull-down samples compared to input samples as peaks. Analysis of peak distribution under differentiated conditions were performed with the bedtools software package for genome arithmetic. Data were visualized with deepTools.

We determined that the type III latency EBV genome establishes a complex network of intragenomic interactions and that PARP1 inhibition alters viral gene expression as well as the occupancy of both CTCF and cohesin chromatin looping factors. We also noted that the viral genes most significantly dysregulated in expression tended to correlate to the regions where intragenomic interactions were altered after PARP inhibition (Fig. 4), raising the possibility that EBV chromatin conformation impacts the transcription of viral genes. To establish a correlation between changes in viral transcripts and changes in EBV 3D structure, we compared the frequency of DNA–DNA interactions from our HiC dataset to the number of reads per region in the EBV genome (forward and reverse) from our RNA-seq data set (Fig. 4). In the top panel labeled “HiC Analysis”, the blue arcs represent DNA–DNA interactions that occur with higher frequency in the control samples (no olaparib treatment) between the two regions it connects. The red arcs represent DNA–DNA interactions that occur with higher frequency after olaparib treatment. Finally, the black arcs are chromatin loops that remain unchanged between both treatment groups. In the middle panel labeled “RNA-seq Analysis”, the blue peaks represent transcripts that are more abundant in the control sample (no olaparib treatment) and the red peaks represent transcripts that are more abundant after olaparib treatment (Fig. 4, middle panel). Starting at the beginning of the linearized episome, we observed that the red peaks along the RNA-seq tracks align perfectly with increased intragenomic contacts at the origin of replication, OriP. This corroborates previous findings from our group and others showing that knockdown of PARP1 regulates OriP function^9,42^. Moving forward along the genome, we also observed high consecutive blue peaks of RNA reads within the EBNA transcripts, which are shown to be downregulated in our RNA-seq data (Fig. 2D), directly aligned with numerous blue arcs (chromatin loops that are more frequent in the untreated EBV episome, Fig. 4). The lytic BMRF2 and BALF3 genes, which are shown to be overexpressed in olaparib treated LCLs, also have heightened RNA-seq reads that can be directly correlated to increased red arcs in the HiC data. The latent genes EBNA3B/3C conversely are under-expressed in olaparib treatment and align with chromatin binding events that are more frequent in the control genome. Finally, though the full-length RPMS1 transcript is downregulated in olaparib treated LCLs, there are a higher number of RNA reads as displayed by the red peak on the RNA-seq track (Fig. 4) that correlates with increased intragenomic contacts at the same RPMS1 transcript region. Though this seems contradictory, we observed that one of the seven tested miRNAs spliced from RPMS1 is significantly upregulated (Supplementary Fig. 4). The discrepancy between our RNA-seq data and RT-qPCR quantification of miRNA could be due to the fact that miRNAs are filtered from the RNA samples during purification before sequencing and therefore they are disregarded during data analysis. Overall, these results demonstrate that in type III latency infected EBV+ B-cells, chromatin conformation is altered after PARP inhibition, and the changes in chromatin looping correlate with dysregulation of viral genes at those regions.

### Altered CTCF and Cohesin occupancy correlates with changes in EBV chromatin looping after PARP inhibition

Once we established that a correlation exists between changes in EBV chromatin architecture and viral gene expression after PARP inhibition, we investigated whether changes in viral chromatin loops in olaparib treated cells involved EBV genomic regions that are occupied by CTCF and cohesin. To determine the role of CTCF/cohesin occupancy in EBV chromatin conformation, we compared changes in their binding via ChIP-seq to our HiC and RNA-seq data as described above (Fig. 4, lower panel). In general, we observed viral chromatin loops occurring between regions where at least one “end” of the chromatin loop is associated with CTCF/cohesin. Interestingly, we noted that the region between 100 and 130 kb forms short chromatin loops in the absence of CTCF and cohesin binding (Fig. 4, lower panel). When we compared changes in chromatin looping and CTCF/RAD21 binding (i.e., loss of CTCF occupancy and increase of RAD21 occupancy), we found that seven regions of the viral genome had altered chromatin architecture correlating with loss of CTCF and increase of RAD21 occupancy after PARP inhibition (Fig. 4, lower panel, green stars). We also noted that changes in CTCF/RAD21 binding result in either the formation or the disruption of chromatin loops. The changes in chromatin looping due to altered CTCF/cohesin occupancy after PARP inhibition correlated to dysregulated transcription of the viral genes within the same regions (Fig. 4, lower panel, green stars). Specifically, we observed that PARP inhibition results in the disruption of the chromatin loops existing upstream of the EBER microRNA encoding region located at 6.6 kb, coinciding with changes in CTCF/RAD21 occupancy. However, after PARP inhibition we observed that the same region at 6.6 kb now establishes a new intragenomic interaction with a region located around 35 kb of the viral genome and that both regions show changes in CTCF/RAD21 occupancy (Fig. 4, lower panel, green stars). These results demonstrate that CTCF and cohesin aid in the organization of EBV episomal structure and that their function is regulated through PARP1 activity.

### Global cohesin abundance and localization is unaffected by PARP inhibition

**Fig. 5:**
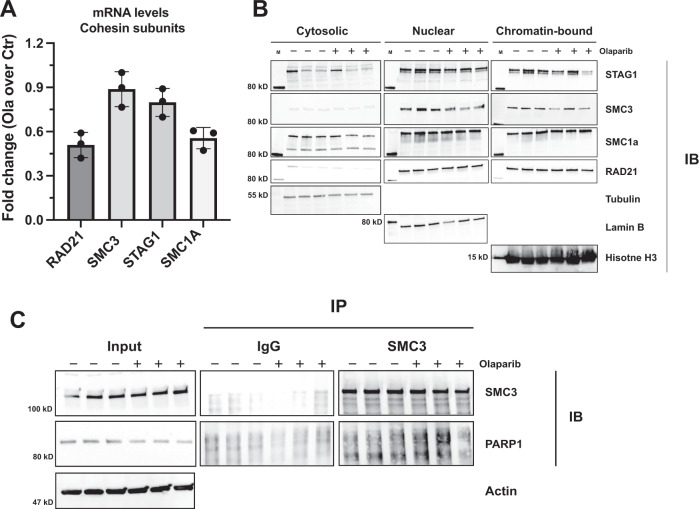
Cohesin expression and localization are unchanged by PARP inhibition. **A** RT-qPCR of each cohesin component, each normalized to 18S, respectively, with and without olaparib treatment in LCL. Data displayed as fold change in expression of olaparib treated LCLs divided by control. The data presented is an average of three independent experiments. Source data are provided as a Source Data file. **B** Western blots of all cohesin components were independently collected from three biological replicates of control or 2.5 µM olaparib treated LCL after subcellular fractionation. Source data are provided as a Source Data file. **C** Immunoprecipitation (IP) of SMC3 component of cohesin in three biological replicates of LCLs, with or without PARP inhibition. Input lysate was collected before IP and ran along IP material for SMC3 and PARP1 western blots. Source data are provided as a Source Data file.

Our data indicate that PARP1 activity regulates EBV chromatin conformation and viral gene expression by modulating CTCF and cohesin occupancy across the viral genome. To ensure the increased cohesin binding frequency observed after PARP inhibition is not merely a function of increased cohesin expression, we performed RT-qPCR on three biological replicates of each treatment group (Fig. 5A). We found no differences in the expression of the cohesin subunit genes before or after PARP inhibition. Next, we assessed whether PARP inhibition affects the protein levels or the subcellular localization of each cohesin component by western blot analysis after subcellular fractionation. We found that for each cohesin component, the protein abundance and localization were unaffected by PARP inhibition (Fig. 5B). As the chromatin-bound fraction showed no difference in total cohesin binding for any of the protein complexes after PARP inhibition, we argue that these results indicate that PARP inhibition is stabilizing cohesin binding at specific genomic sites on the EBV genome resulting in a higher frequency of reads observed in the cohesin binding profile. However, it is also of note that the majority of chromatin-bound cohesin in the assay is from the human genome, indicating that total cohesin binding is unaffected across both genomes, or that a global decrease in cohesin binding across the EBV genome was masked by the abundance of cohesin binding on the human genome. Finally, to determine if cohesin binding is increased at the assessed areas on the EBV genome due to an increase in its affinity for PARP1 interaction after olaparib treatment, we performed an immunoprecipitation assay with SMC3 antibody in three biological replicates of type III LCLs, with or without olaparib treatment (Fig. 5C). When immunoblotted for PAPR1, we found no difference in cohesin/PARP1 interaction between treatment groups. Taken together, this data indicates that the increased cohesin binding observed across the EBV genome after PARP enzymatic inhibition may be a function specific to the EBV genome and that it is likely, not due to an increased affinity for PARP1/cohesin binding.

### Chromatin folding brings EBV regulatory loci in close proximity

Our HiC analysis reveals at the genome-wide level the intricate map of physical contacts existing between different regions of the EBV genome. From this, we were able to illustrate that the type III latency EBV genome is organized into a complex network of DNA–DNA contacts that are partially regulated by PARP activity. We next decided to use the DNA–DNA contact interactions determined by our HiC analysis to delineate a 3D model of the EBV episome as folded into the host cell nucleus, before and after PARP inhibition.

**Fig. 6:**
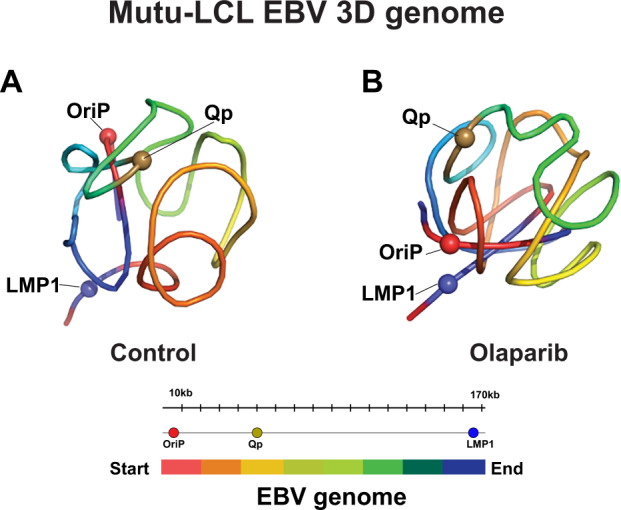
Chromatin regulatory region localization is altered by PARP inhibition. PASTIS model displaying control type III EBV genome (**A**) or olaparib treated (**B**) with the color-coded legend of EBV genome. The model was derived from HiC matrices.

To infer how the EBV episome is folded, we used the PASTIS software package^43,44^ to generate the 3D model of the EBV genome for type III latency from our HiC datasets (Fig. 6). The models of the EBV episome before and after PARP inhibition are dissimilar. In control cells, the origin of viral replication OriP, the EBNAs promoter Cp, and the latent promoter Qp, cluster together (Fig. 6A). The 3D model also illustrates the OriP region separated from the divergent promoter region of LMP1, which appeared to interact with OriP in our HiC analysis and in our previous work^25^. This discrepancy between the 3D model and the HiC data could be due to a lack of accuracy by the PASTIS software to model circular regions of DNA such as viral episomes.

The 3D model of the EBV genome after PARP inhibition shows OriP and Qp are no longer colocalized (Fig. 6B). In addition, OriP and the LMP1 promoter are now brought in close proximity unlike in the control genome. Overall, the inferred model of the EBV chromosome from type III latently infected cells shows that important regulatory region of EBV cluster together as a consequence of EBV episome folding. Moreover, our 3D models underscore the importance of PARP activity in the folding of EBV episome by demonstrating the effect of PARP inhibition on 3D chromatin structure.

## Discussion

In this work, we set out to determine if 3D structure of the EBV episome (1) differs between latency types, (2) is dependent upon PARP1 enzymatic activity, and (3) can be correlated to latency-type specific gene expression. To answer these questions, we have generated a complete 3D model of the EBV episome in type I and type III latency states, as assayed through statistical modeling of in situ HiC matrices from two biological replicates. Interestingly, despite being isogenic with respect to the EBV genome, the two latency types displayed vastly different structures of viral episome, with type III having significantly more intragenomic contacts than type I. These results demonstrate that EBV episome can be differentially folded in the host nucleus between latency types.

CTCF plays an important role in establishing chromatin loops. However, in the context of EBV, the distinct 3D structures we observed cannot be accounted for by differences in CTCF binding between latency types, as type I and type III CTCF profiles were found to be highly similar. Taken together, though CTCF has been shown to play a critical role in the establishment and maintenance of latency repertoires^11,20,21,45^, our results indicate that CTCF–chromatin binding cannot account for the distinct profiles of the EBV episome found via global HiC analysis. Since cohesin is known to colocalize with CTCF across the type III EBV genome^18,19^, we additionally examined the cohesin binding profiles of the same cell lines, with and without PARP inhibition. As with CTCF, we noted a nearly identical binding profile between latency types. However, cohesin occupancy was increased across the type III genome after PARP inhibition while CTCF binding was decreased. As no relationship has previously been published to the best of our knowledge regarding PARP1 regulation of cohesin-chromatin binding outside of double-strand break repair^46^, we were excited by this finding establishing a functional link between PARP activity and cohesin/CTCF interaction. Future experiments are needed to determine the mechanism by which PARP regulates cohesin-chromatin binding. Based on our data, numerous hypotheses can be considered including the direct PARylation of cohesin impeding ATPase-dependent motility or an indirect effect of PARP inhibition on other chromatin effector molecules.

The future mechanistic examination could also expand the current view of cohesin–CTCF dynamics, as the widely accepted loop-extrusion model implies that lower CTCF binding frequency would prevent the accumulation of cohesin at those sites. With this in mind, increased cohesin binding after olaparib treatment seems contradictory. However, work in CTCF-depleted fibroblasts has demonstrated that cohesin-chromatin binding is not prevented by the absence of CTCF, but its localization is rather centralized to active transcriptional start sites^47^. Cohesin is also known to bind chromatin independent of CTCF in a cell-type-specific and highly conserved pattern, generally in promoter and enhancer regions of active genes^48^. Most work studying post-translational modifications of cohesin and their impacts on DNA-binding affinity is in the context of sister-chromatid cohesion and DNA damage. In these instances, SUMOylation of RAD21 enhances cohesin-chromatin binding affinity^49^, while phosphorylation of SA1 yeast homolog Scc1 induces dissociation from DNA^50^. Taken together, there is precedence that not only is cohesin found to bind independently of CTCF, but its chromatin binding is also known to be influenced by post-translational modifications. For these reasons, we believe that our data demonstrating that PARylation increases cohesin stability on the EBV episome despite lower CTCF binding would be an important contribution to the field. However, one potential caveat of our observations is the limited size of the EBV genome (~170 kb) which may not fully recapitulate the interactions between CTCF and cohesin observed in mammalian genomes at megabase scales^14,28,38^. Nevertheless, our results indicate that in the context of relatively small loops, PARP1 activity may be critical to the regulation of cohesin binding to DNA.

As PARP1’s role in chromatin modification has been well established in various systems^8,51,52^ including EBV^9,42^, we performed an additional HiC assay on the same cell lines after PARP inhibition and found that both the type I and type III genome had a decrease in total unique intragenomic interactions. However, while type I exclusively lost chromatin loops, type III gained new chromatin contacts not seen in the control genome (Fig. 1). When viewing the raw dataset explicitly outlining the two fragments which ligated during the HiC assay with an additional statistical parameter of FDR ≤ 0.05, we see that the type III genome maintains the same number of chromatin loops (Supplementary data 1). The 17 DNA-DNA interactions are not static between the two groups though as the identity of the fragments are distinct between treatment and control, underscoring the central point that PARP enzymatic inhibition destabilizes the 3D EBV genome structure.

Unexpectedly, we observed differences between our HiC analysis and the chromatin loops identified in a previous study using the same isogenic type I and type III cell lines^27^. This could be explained by differences in methodology between in vitro chromatin conformation capture assays (3C) and in situ HiC. HiC provides a global, unbiased view of all intragenomic interactions within the episome, but only at an average 5 kb resolution in this study. Because of this, the relatively local interaction previously identified in Mutu-LCL between OriP and Cp may be too close to be identified in HiC analysis. Our analysis did agree with another prior publication, however, showing strong intragenomic interactions between the region just before OriP and the LMP1/LMP2a transcript region^25^. In type I latency, the chromatin loop previously observed between the OriP and Qp was also not detected in our HiC analysis. As 3C requires longer crosslinking time, predetermined primer sets, and many more PCR cycles than are required for HiC analysis, it is possible that prior studies may have reported chromatin loops occurring at very low frequencies. It is also possible that the process of lysing the nuclei and incubating the contents together allow for chromatin binding events that would not be possible using an in situ approach.

This work also establishes the functional effect of altered chromatin looping after PARP inhibition on EBV gene expression. The RNA-seq analysis performed with or without PARP enzymatic inhibition on the same cell lines shows that viral genes expression is altered after olaparib treatment. Type I cells displayed significant dysregulation of only three viral genes while type III showed altered expression of ten viral genes. For both cell types, lytic genes were upregulated, consistent with prior work demonstrating that PARP1 knockdown increases viral copy number and lytic reactivation^10,42,53^. In LCLs, genes indicative of type III latency, namely the EBNAs, were significantly downregulated, corroborated by earlier work illustrating the downregulation of EBNA2 in the same cell line after PARP inhibition^10^. These data together with observed changes in the viral 3D chromatin structure after PARP inhibition indicate that PARP1 aids in the maintenance of latency programs and prevention of lytic gene expression, especially in type III latency.

We were also interested in the correlation between increased cohesin binding and increased RNA reads at the same regions. While demonstrating a direct causal relationship between gene expression and particular chromatin interactions would require the mutation/deletion of cohesin binding sites, we are excited that this global, correlational study provides the rationale for future studies of EBV genome structure and maintenance of latency repertoires. Our findings are of particular interest for other DNA viruses as previous work has shown that CTCF/cohesin has a major role in the regulation of transcription and latency maintenance in HSV1^54–58^, KSHV^59–63^, HCMV^64^, HPV^65–67^, and HTLV-1^68^. In these viruses, it could be interesting to determine whether PARP1 similarly regulates the viral epigenome through CTCF or cohesin. Outside of virology, this work reports PARP1’s role as a regulator of 3D chromatin structure on a global scale utilizing the EBV episome as a model.

Finally, we would be remiss to not include the major limitations of this study as points of consideration. The major conclusions of this project were outlined by our H–C analysis and models made from EBV chromatin extracted from populations of five-million cells per treatment group. It has long been established that EBV episome copy number varies not only by strain and viral lifecycle but also by genetic variation between host B-cells^69–71^. Because of this, we do wonder whether different EBV strains exhibiting the same latency type would have similar episomal conformation as those we have elucidated in this project. In addition, it is interesting to speculate if the same strain of EBV in a different donor B-cell background would display the same chromatin conformation. On a technical level, the in situ HiC method utilized in this paper can only generate an inferred 3D chromatin structure from a population dataset, meaning these results cannot determine if individual episomes within the same human B-cell display different chromatin conformations. The models generated here can only illustrate the most frequently occurring DNA-DNA interactions as a generalized chromatin conformation for the population. While the limitations outlined here should certainly be taken into consideration, we are still excited to report that isogenic EBV genomes displaying different latency types have vastly different chromatin conformations that are regulated by PARP1 enzymatic activity.

## Methods

### Cell culture and drug treatment

The type I latency “Mutu I” Burkitt Lymphoma cell line and the type III latency lymphoblastoid cell line “Mutu-LCL” were maintained in a humidified incubator at 37 °C and 5% CO_2_ in RPMI 1640 medium supplemented with 1% penicillin/streptomycin and 15% fetal bovine serum. Treatment with PARP inhibitor olaparib (Selleck Chemicals, Catalog No. S1060) was given 72 h before collection at a concentration of 2.5 µM. PARP inhibition was validated via PAR ELISA as per the manufacturer’s protocol (PARP In Vivo Pharmacodynamic Assay II, Trevigen, Catalog No. 4520-096-K). The relative luminescence was measured on the EnVision 2104 Multilabel Reader (PerkinElmer). PDD00017273 treatment (Selleck Chemicals, Catalog No. S8862) started 72 h before collection, with redosing every 24 h, at concentrations varying from 0 to 50 µM. PARG inhibition was validated by western blot against PAR (Abcam, Product No. ab14459). For chromatin immunoprecipitation assay cells were treated with 2.5 µM PDD00017273 for 72 h.

### Antibodies

The following antibodies at the indicated dilutions or concentrations were used: RAD21: Abcam Product No. ab992, WB: 1:1000, IP: 2.5 μg, ChIP-seq, 5 μg; STAG1: Abcam Product No. ab4457, WB: 1:1000, IP: 2.5 μg; SMC1: Abcam Product No. ab9262, WB: 1:1000, IP: 2.5 μg; SMC3: Abcam Product No. ab9263, WB: 1:1000, IP: 2.5 μg, ChIP 2.5 μg; CTCF: Active Motif Catalog No. 61311, WB: 1:2000, ChIP-seq: 5 μg; IgG: Jackson ImmunoResearch, Product No. 111-005-003, IP: 2.5 μg; βtubulin: Abcam ab6046, WB: 1:1000; Lamin B1: Abcam ab16048, WB: 1:1000; Histone H3: Abcam ab1791, WB: 1:1:000; PARP1: Abcam ab227244, WB: 1:1000; IP: 2.5 μg; PARP1 Active Motif, No: 39561, ChIP: 10 μl per ChIP; PAR: Abcam Product No. ab14459 WB: 1:200; PARG: Abcam Product No. ab169639 WB: 1:2000; Actin: Sigma-Aldrich, No. A2066, WB: 1:200.

### Chromatin immunoprecipitation assays

#### Chromatin immunoprecipitationsequencing (ChIP-seq)

After 72 h incubation with or without olaparib, 25 million cells per immunoprecipitation were collected and fixed with 1% formaldehyde to preserve all DNA-protein interactions for 15 min and then quenched with 0.25 M glycine for 5 min on ice. Pellet was resuspended after centrifugation in 10 mL each of a series of three lysis buffers before fragmentation in Qsonica sonicator (90AMP, 25 sonication cycles, 30 s on/30 s off) to generate chromatin fragments roughly 200–500 bp in size. Chromatin was centrifuged to clear debris and a sample of this cleared chromatin (1% of total material) was kept as standard input for comparison against immunoprecipitations. Chromatin was incubated rotating at 4° overnight with 25 µg of antibody against RAD21 (Abcam, Product No. ab992). Chromatin–antibody complexes were precipitated using 50 µL of Dynabeads Protein A (ThermoFisher, Product No. 10001D) incubated rotating at 4° overnight. Beads were collected on a magnetic rack and resuspended in 1 mL modified RIPA buffer and were rotated for one minute at room temperature. Beads were collected on the magnetic rack, the wash was repeated four additional times, with the final wash in TE buffer. After the final wash step, beads were resuspended in 60 µL elution buffer and incubated for 15 min in a 65° thermomixer at max RPM. Eluant was collected and 0.2 M NaCl was added to each sample (including input chromatin). All samples were incubated in 65° water bath overnight to reverse crosslink. DNA was purified using Promega Wizard SV Gel and PCR Clean-up Kit (Product No. A9285). Libraries for sequencing were made NEBNext^®^ Ultra^™^ II DNA Library Prep Kit (New England Biolabs, Product No. E7103) and sequenced on the hiseq2500 (Illumina).

#### ChIP-seq analysis

Reads were mapped against the human gammaherpesvirus 4 (HHV4) NC_007605.1 genome assembly using bowtie2^26^. We used MACS2^72,73^ software packages to call reads enrichment in pull-down samples compared to input samples as peaks. Analysis of peak distribution under differentiated conditions was performed with the bedtools software package for genome arithmetic^74^, and for data visualization we used deepTools^75^. RNA-seq and ChIP-seq data were deposited for public access at Gene Expression Omnibus (GEO accession number: GSE159837).

#### Chromatin immunoprecipitation (ChIP)

After 72 h incubation with or without olaparib, 1 million cells per immunoprecipitation were collected and fixed with 1% formaldehyde to preserve all DNA–protein interactions for 15 min and then quenched with 0.125 M glycine for 5 min on ice. After centrifugation, the pellet was resuspended in 120 µL lysis buffer and sonicated using Covaris S2 (Duty Cycle: 10%, Intensity: 5, Cycles/Burst: 200, Time: 120 s (60 s *2 cycles) to generate chromatin fragments roughly 200–500 bp in size. A sample of “input chromatin” was collected at this point as a standard for comparison against immunoprecipitations (1% of total material). Chromatin was then incubated overnight rotating at 4° with 2.5 µg of antibody against each cohesin component, CTCF, or IgG, respectively (RAD21: Abcam Product No. ab992, STAG1: Abcam Product No. ab4457, SMC1: Abcam Product No. ab9262, SMC3: Abcam Product No. ab9263, CTCF: Active Motif Catalog No. 61311, IgG: Jackson ImmunoResearch, Product No. 111-005-003). Chromatin–antibody complexes were precipitated via 2-h incubation with 50 µL of Dynabeads Protein A rotating at 4°. Beads were washed with 1 mL of a series of three wash buffers, followed by two washes with TE buffer. Washed beads were resuspended in 150 µL SDS-TE and incubated for 15 min in a 65° thermomixer at max RPM. Beads were cleared on a magnetic rack and eluant was incubated in a 65° water bath overnight to reverse DNA–protein crosslink. Proteinase K (Ambion, Product No. AM2546) was then added at 20 mg/mL and incubated in a 55° water bath for 3 h. DNA was then recovered using Promega Wizard SV Gel and PCR Clean-up Kit (Product No. A9285). Real-time PCR was performed with a master mix containing 1× Maxima SYBR Green (Thermoscientific, REF No. K0223) 0.25 μM primers and 1% of ChIP or input DNA per well. Data were analyzed by the ΔΔCT method relative to DNA input and normalized to the IgG control. Primer sequences are provided in Supplementary Data 3.

### HiC

After 72 h incubation with or without olaparib, five million cells per condition were collected for in situ HiC^28^, with minor modifications. Libraries of total ligation products were produced using Ultralow Library Systems V2 (Tecan Genomics, part No. 0344NB-32) as per manufacturer’s protocol with minor modifications. Purified libraries were then enriched for only EBV genome ligation products using myBaits enrichment kit as per manufacturer’s protocol. Enriched libraries were sequenced using hiseq2500 (Illumina) with paired-end 75 bp read length. Complete protocol with all minor alterations will be happily supplied by the corresponding author per request.

*Processing HiC data*. HiC data were processed as described previously^76^. Briefly, 75-bp paired reads were separately aligned to the EBV genome (NC_007605.1) using Bowtie2 (version 2.2.9) with iterative alignment strategy^76^. Redundant paired reads derived from a PCR bias reads aligned to repetitive sequences, and reads with low mapping quality (MapQ < 30) were removed. Reads potentially derived from self-ligation and undigested products were also discarded. EBV genome was divided into 5 kb windows with 1 kb sliding. Raw contact matrices were constructed by counting paired reads assigned to two 5 kb windows. HiC biases in contact matrices were corrected using the ICE method^77^. The ICE normalization was repeated 30 times. Significant associations were determined based on the distance between two 5 kb windows, all combinations were categorized into 20 groups. We assumed the HiC score as Poisson distribution with a parameter λ matching the mean score. We then assigned a *p* values for each group and applied an FDR correction for multiply hypotheses^78^. FDR < 0.05 were defined as significant associations. Significant associations were plotted as circos graph using the circlize package (version 0.3.3) of R (version 3.6.1).

### RT-qPCR

Five million cells were collected for three biological replicates of both Mutu-LCL untreated and Mutu-LCL 2.5 μM olaparib treated for 72 h. Cells were centrifuged, washed twice with PBS, and resuspended in trizol. RNA was extracted using chloroform and isopropanol. After DNAse treatment, cDNA was prepared using SuperScript^™^ III First-Strand Synthesis System (Invitrogen, Catalog No. 18080051). Real-time PCR was then performed with a master mix containing 1X Maxima SYBR Green (Thermoscientific, REF No. K0223) 0.25 μM primers and 25% cDNA. Data were analyzed by the ΔCT method relative to 18S-ribosomal subunit control. Primer sequences are provided in Supplementary Data 3.

### Subcellular fractionation and western blotting

Three biological replicates of 10 million Mutu-LCL per treatment group (with or without 2.5 μM olaparib for 72 h) were prepared using the Subcellular Protein Fractionation Kit for Cultured Cells from Thermo Scientific (Catalog No. 78840). Respective proteins of interest were probed via western blot using antibodies listed previously for chromatin immunoprecipitations. Samples were normalized to the following loading controls: β-tubulin (Abcam ab6046) for cytosolic extracts, lamin B1 (Abcam ab16048) for nuclear soluble extracts, and histone H3 (Abcam ab1791) for chromatin-bound extracts.

### Co-immunoprecipitation protein assays

For SMC3-immunoprecipitation (IP) assays, 10 million LCLs, with or without 72-h 2.5 μM olaparib treatment, were collected for each IP and resuspended in 1 mL of RIPA buffer with protease/phosphatase inhibitor cocktail (Thermo Scientific). Before the addition of 10 μg of either SMC3 (Abcam, ab9263) or normal rabbit IgG (Jackson, 111-005-003), 50 μL of cell lysate was collected and kept as input material. Cell lysates were incubated with respective antibodies for 1 h at room temperature, rotating, after which 30uL of protein A magnetic beads (Invitrogen, 10001D) were added. The mixture was left to incubate overnight at 4°, rotating. The beads were then separated with a magnetic rack and washed three times in RIPA buffer with protease/phosphatase inhibitor, each for 10 min in a 4° thermomixer at 1000 rpm. The beads were then boiled at 95° for 8 min in 50 μL 2× laemmli buffer, with half of the volume ran on an immunoblot for SMC3 and half for PARP1 (Abcam, ab227244) as described above. Densitometry analysis was performed on Invitrogen iBright Analysis Software, with signal density/area from IgG control lanes subtracted from IP lanes. IgG normalized IP signal was then normalized to input signal density/area. Data shown are representative of three independent co-IP assays, averaged.

### RNA-sequencing

Five million cells each for two biological replicates were collected per control and olaparib treatment groups for RNA sequencing. RNA was purified using the PureLink RNA Mini Kit from Thermofisher (cat no. 12183018 A). Libraries for sequencing were made using NEBNext Ultra^™^ Directional RNA Library Prep Kit for RNAseq (Cat #E4720L) and sequenced on the hiseq2500 (Illumina).

*RNA-seq analysis*. Sequenced reads were aligned to the human gammaherpesvirus 4 (HHV4) NC_007605.1 genome assembly using the STAR software suite^79^. To determine differential gene expression, we used the EdgeR software suite^43^. Genes with 0 reads across all samples were excluded. Genes with 2-fold change and *q* < 0.01 after correction for multiple testing (FDR) were considered as significantly differentially expressed. We used R software packages (CRAN project and Bioconductor) for downstream analysis of RNA-seq such as hierarchical clustering and PCA. Ingenuity Pathway Analysis (Qiagen) was used to determine functional gene enrichment.

### Reporting summary

Further information on research design is available in the Nature Research Reporting Summary linked to this article.

## Supplementary information

Supplemental Information

Description of additional Supplementary File

Supplementary Data 1

Supplementary data 2

Supplementary data 3

Reporting Summary

## Data Availability

The data that support this study are available from the corresponding author upon reasonable request. The data for the HiC assay, ChIP-seq assay for CTCF, SMC3 and RAD21, RNA-seq for Mutu I and Mutu-LCL before and after treatment with Olaparib have been deposited in the Gene Expression Omnibus database, GEO, under the following accession codes: GSE160973, GSE159834, GSE159836, GSE159837. ChIP-seq for CTCF in LCL cells before and after PARP inhibition was obtained by publicly available sequencing datasets GSE115829, generated in our previous work^10^. Source data are provided with this paper.

## Source data

Source Data

## References

[1] Price AM, Luftig MA (2015). To Be or Not IIb: a multi-step process for Epstein-Barr virus latency establishment and consequences for B cell tumorigenesis. PLOS Pathog..

[2] Hsu JL, Glaser SL (2000). Epstein-Barr virus-associated malignancies: epidemiologic patterns and etiologic implications. Crit. Rev. Oncol. Hematol..

[3] Thorley-Lawson DA, Gross A (2004). Persistence of the Epstein-Barr virus and the origins of associated lymphomas. N. Engl. J. Med..

[4] Parkin DM (2006). The global health burden of infection-associated cancers in the year 2002. Int. J. Cancer.

[5] Ferlay J (2010). Estimates of worldwide burden of cancer in 2008: GLOBOCAN 2008. Int. J. Cancer.

[6] Khan G, Hashim M (2014). Global burden of deaths from Epstein-Barr virus attributable malignancies 1990-2010. Infect. Agents Cancer.

[7] Farrell PJ (2019). Epstein-Barr virus and cancer. Annu. Rev. Pathol..

[8] Gupte R, Liu Z, Kraus WL (2017). PARPs and ADP-ribosylation: recent advances linking molecular functions to biological outcomes. Genes Dev..

[9] Lupey-Green LN (2017). PARP1 restricts Epstein Barr Virus lytic reactivation by binding the BZLF1 promoter. Virology.

[10] Lupey-Green LN (2018). PARP1 stabilizes CTCF binding and chromatin structure to maintain Epstein-Barr virus latency type. J. Virol..

[11] Tempera I, Wiedmer A, Dheekollu J, Lieberman PM (2010). CTCF prevents the epigenetic drift of EBV latency promoter Qp. PLoS Pathog..

[12] Martin KA, Lupey LN, Tempera I (2016). Epstein-Barr virus oncoprotein LMP1 mediates epigenetic changes in host gene expression through PARP1. J. Virol..

[13] Phillips JE, Corces VG (2009). CTCF: master weaver of the genome. Cell.

[14] Rowley MJ, Corces VG (2018). Organizational principles of 3D genome architecture. Nat. Rev. Genet..

[15] Ong C-T, Bortle KV, Ramos E, Corces VG (2013). Poly(ADP-ribosyl)ation regulates insulator function and intrachromosomal interactions in Drosophila. Cell.

[16] Farrar D (2010). Mutational analysis of the poly(ADP-ribosyl)ation sites of the transcription factor CTCF provides an insight into the mechanism of its regulation by poly(ADP-ribosyl)ation. Mol. Cell. Biol..

[17] Zampieri M (2012). ADP-ribose polymers localized on Ctcf-Parp1-Dnmt1 complex prevent methylation of Ctcf target sites. Biochem. J..

[18] Holdorf, M. M., Cooper, S. B., Yamamoto, K. R. & Miranda, J. J. L. Occupancy of chromatin organizers in the Epstein-Barr virus genome. *Virology*10.1016/j.virol.2011.04.004 (2011). 10.1016/j.virol.2011.04.004 PMC3808970 21550623

[19] Arvey A (2012). An atlas of the Epstein-Barr virus transcriptome and epigenome reveals host-virus regulatory interactions. Cell Host Microbe.

[20] Chau CM, Zhang X-Y, McMahon SB, Lieberman PM (2006). Regulation of Epstein-Barr virus latency type by the chromatin boundary factor CTCF. J. Virol..

[21] Day L (2007). Chromatin profiling of Epstein-Barr virus latency control region. J. Virol..

[22] Davidson, I. F. et al. DNA loop extrusion by human cohesin. *Science*10.1126/science.aaz3418 (2019). 10.1126/science.aaz3418 31753851

[23] Kagey MH (2010). Mediator and cohesin connect gene expression and chromatin architecture. Nature.

[24] Millau J-F, Gaudreau L (2011). CTCF, cohesin, and histone variants: connecting the genome. Biochem. Cell Biol..

[25] Chen H-S (2014). Epigenetic deregulation of the LMP1/LMP2 locus of Epstein-Barr virus by mutation of a single CTCF-cohesin binding site. J. Virol..

[26] Langmead B, Salzberg SL (2012). Fast gapped-read alignment with Bowtie 2. Nat. Methods.

[27] Tempera I, Klichinsky M, Lieberman PM (2011). EBV latency types adopt alternative chromatin conformations. PLoS Pathog..

[28] Rao SS (2014). A 3D map of the human genome at kilobase resolution reveals principles of chromatin looping. Cell.

[29] Menear KA (2008). 4-[3-(4-cyclopropanecarbonylpiperazine-1-carbonyl)-4-fluorobenzyl]-2H-phthalazin- 1-one: a novel bioavailable inhibitor of poly(ADP-ribose) polymerase-1. J. Med. Chem..

[30] Johannsen E (2004). Proteins of purified Epstein-Barr virus. Proc. Natl Acad. Sci. USA.

[31] Chiu SH (2014). Epstein-Barr virus BALF3 has nuclease activity and mediates mature virion production during the lytic cycle. J. Virol..

[32] Paulsen SJ, Rosenkilde MM, Eugen-Olsen J, Kledal TN (2005). Epstein-Barr virus-encoded BILF1 is a constitutively active G protein-coupled receptor. J. Virol..

[33] Beisser PS (2005). The Epstein-Barr virus BILF1 gene encodes a G protein-coupled receptor that inhibits phosphorylation of RNA-dependent protein kinase. J. Virol..

[34] Zuo J (2009). The Epstein-Barr virus G-protein-coupled receptor contributes to immune evasion by targeting MHC class I molecules for degradation. PLoS Pathog..

[35] Gill MB, Edgar R, May JS, Stevenson PG (2008). A gamma-herpesvirus glycoprotein complex manipulates actin to promote viral spread. PLoS ONE.

[36] Xiao J, Palefsky JM, Herrera R, Berline J, Tugizov SM (2008). The Epstein-Barr virus BMRF-2 protein facilitates virus attachment to oral epithelial cells. Virology.

[37] Matveeva E (2016). Involvement of PARP1 in the regulation of alternative splicing. Cell Discov..

[38] Zhang K, Li N, Ainsworth RI, Wang W (2016). Systematic identification of protein combinations mediating chromatin looping. Nat. Commun..

[39] Sonoda E (2001). Scc1/Rad21/Mcd1 is required for sister chromatid cohesion and kinetochore function in vertebrate cells. Dev. Cell.

[40] James DI (2016). First-in-class chemical probes against poly(ADP-ribose) glycohydrolase (PARG) inhibit DNA repair with differential pharmacology to olaparib. ACS Chem. Biol..

[41] Murai J (2014). Stereospecific PARP trapping by BMN 673 and comparison with olaparib and rucaparib. Mol. Cancer Therap..

[42] Tempera I (2010). Regulation of Epstein-Barr virus OriP replication by poly(ADP-ribose) polymerase 1. J. Virol..

[43] Robinson MD, McCarthy DJ, Smyth GK (2010). edgeR: a Bioconductor package for differential expression analysis of digital gene expression data. Bioinformatics.

[44] Varoquaux N, Ay F, Noble WS, Vert JP (2014). A statistical approach for inferring the 3D structure of the genome. Bioinformatics.

[45] Hughes DJ (2012). Contributions of CTCF and DNA methyltransferases DNMT1 and DNMT3B to Epstein-Barr virus restricted latency. J. Virol..

[46] O’Neil NJ, van Pel DM, Hieter P (2013). Synthetic lethality and cancer: cohesin and PARP at the replication fork. Trends Genet..

[47] Busslinger GA (2017). Cohesin is positioned in mammalian genomes by transcription, CTCF and Wapl. Nature.

[48] Kojic A (2018). Distinct roles of cohesin-SA1 and cohesin-SA2 in 3D chromosome organization. Nat. Struct. Mol. Biol..

[49] McAleenan A (2012). SUMOylation of the alpha-kleisin subunit of cohesin is required for DNA damage-induced cohesion. Curr. Biol..

[50] Alexandru G, Uhlmann F, Mechtler K, Poupart MA, Nasmyth K (2001). Phosphorylation of the cohesin subunit Scc1 by Polo/Cdc5 kinase regulates sister chromatid separation in yeast. Cell.

[51] Kraus WL, Lis JT (2003). PARP goes transcription. Cell.

[52] Ray Chaudhuri A, Nussenzweig A (2017). The multifaceted roles of PARP1 in DNA repair and chromatin remodelling. Nat. Rev. Mol. Cell Biol..

[53] Mattiussi S (2007). Inhibition of poly(ADP-ribose)polymerase impairs Epstein Barr virus lytic cycle progression. Infect. Agents Cancer.

[54] Lang F (2017). CTCF interacts with the lytic HSV-1 genome to promote viral transcription. Sci. Rep..

[55] Ertel MK, Cammarata AL, Hron RJ, Neumann DM (2012). CTCF occupation of the herpes simplex virus 1 genome is disrupted at early times postreactivation in a transcription-dependent manner. J. Virol..

[56] Washington, S. D., Musarrat, F., Ertel, M. K., Backes, G. L. & Neumann, D. M. CTCF binding sites in the herpes simplex virus 1 genome display site-specific ctcf occupation, protein recruitment, and insulator function. *J. Virol*. 10.1128/JVI.00156-18 (2018). 10.1128/JVI.00156-18 PMC5874429 29437965

[57] Watson, Z. L. et al. In vivo knockdown of the herpes simplex virus 1 latency-associated transcript reduces reactivation from latency. *J Virol*10.1128/JVI.00812-18 (2018). 10.1128/JVI.00812-18 PMC6069208 29875240

[58] Washington, S. D. et al. Depletion of the insulator protein CTCF results in herpes simplex virus 1 reactivation in vivo. *J. Virol.*10.1128/JVI.00173-18 (2018). 10.1128/JVI.00173-18 PMC5952164 29514910

[59] Stedman W (2008). Cohesins localize with CTCF at the KSHV latency control region and at cellular c-myc and H19/Igf2 insulators. EMBO J..

[60] Kang H, Lieberman PM (2009). Cell cycle control of Kaposi’s sarcoma-associated herpesvirus latency transcription by CTCF-cohesin interactions. J. Virol..

[61] Kang H, Wiedmer A, Yuan Y, Robertson E, Lieberman PM (2011). Coordination of KSHV latent and lytic gene control by CTCF-cohesin mediated chromosome conformation. PLoS Pathog.

[62] Li, D., Mosbruger, T., Verma, D. & Swaminathan, S. Complex interactions between cohesin and CTCF in regulation of Kaposi’s Sarcoma-associated herpesvirus lytic transcription. *J. Virol.*10.1128/JVI.01279-19 (2020). 10.1128/JVI.01279-19 PMC6955261 31666380

[63] Li DJ, Verma D, Mosbruger T, Swaminathan S (2014). CTCF and Rad21 act as host cell restriction factors for Kaposi’s sarcoma-associated herpesvirus (KSHV) lytic replication by modulating viral gene transcription. PLoS Pathog..

[64] Martínez FP (2014). CTCF binding to the first intron of the major immediate early (MIE) gene of human cytomegalovirus (HCMV) negatively regulates MIE gene expression and HCMV replication. J. Virol..

[65] Mehta K, Gunasekharan V, Satsuka A, Laimins LA (2015). Human papillomaviruses activate and recruit SMC1 cohesin proteins for the differentiation-dependent life cycle through association with CTCF insulators. PLoS Pathog..

[66] Paris C (2015). CCCTC-binding factor recruitment to the early region of the human papillomavirus 18 genome regulates viral oncogene expression. J. Virol..

[67] Pentland I (2018). Disruption of CTCF-YY1-dependent looping of the human papillomavirus genome activates differentiation-induced viral oncogene transcription. PLoS Biol..

[68] Martinez MP (2019). HTLV-1 CTCF-binding site is dispensable for in vitro immortalization and persistent infection in vivo. Retrovirology.

[69] Mandage R (2017). Genetic factors affecting EBV copy number in lymphoblastoid cell lines derived from the 1000 Genome Project samples. PLoS ONE.

[70] Houldcroft CJ (2014). Host genetic variants and gene expression patterns associated with Epstein-Barr virus copy number in lymphoblastoid cell lines. PLoS ONE.

[71] Davies ML (2010). Cellular factors associated with latency and spontaneous Epstein-Barr virus reactivation in B-lymphoblastoid cell lines. Virology.

[72] Zhang Y (2008). Model-based analysis of ChIP-Seq (MACS). Genome Biol..

[73] Feng J, Liu T, Qin B, Zhang Y, Liu XS (2012). Identifying ChIP-seq enrichment using MACS. Nat. Protoc..

[74] Quinlan AR, Hall IM (2010). BEDTools: a flexible suite of utilities for comparing genomic features. Bioinformatics.

[75] Ramirez F (2016). deepTools2: a next generation web server for deep-sequencing data analysis. Nucleic Acids Res..

[76] Tanizawa H, Kim KD, Iwasaki O, Noma KI (2017). Architectural alterations of the fission yeast genome during the cell cycle. Nat. Struct. Mol. Biol..

[77] Imakaev M (2012). Iterative correction of Hi-C data reveals hallmarks of chromosome organization. Nat. Methods.

[78] Benjamini Y, Hochberg Y (1995). Controlling the false discovery rate: a practical and powerful approach to multiple testing. J. R. Stat. Soc. Ser. B (Methodol.).

[79] Dobin A (2013). STAR: ultrafast universal RNA-seq aligner. Bioinformatics.

