# Supplementary material for: The three-dimensional structure of Epstein-Barr virus genome varies by latency type and is regulated by PARP1 enzymatic activity
Source: Nat Commun. 2022 Jan 17;13:187. doi: 10.1038/s41467-021-27894-1 (PMC8764100; doi:10.1038/s41467-021-27894-1)
Supplement: Supplementary file 1 — Supplemental Information [file 41467_2021_27894_MOESM1_ESM.pdf]

# Supplementary Figures

**A)** **Control**

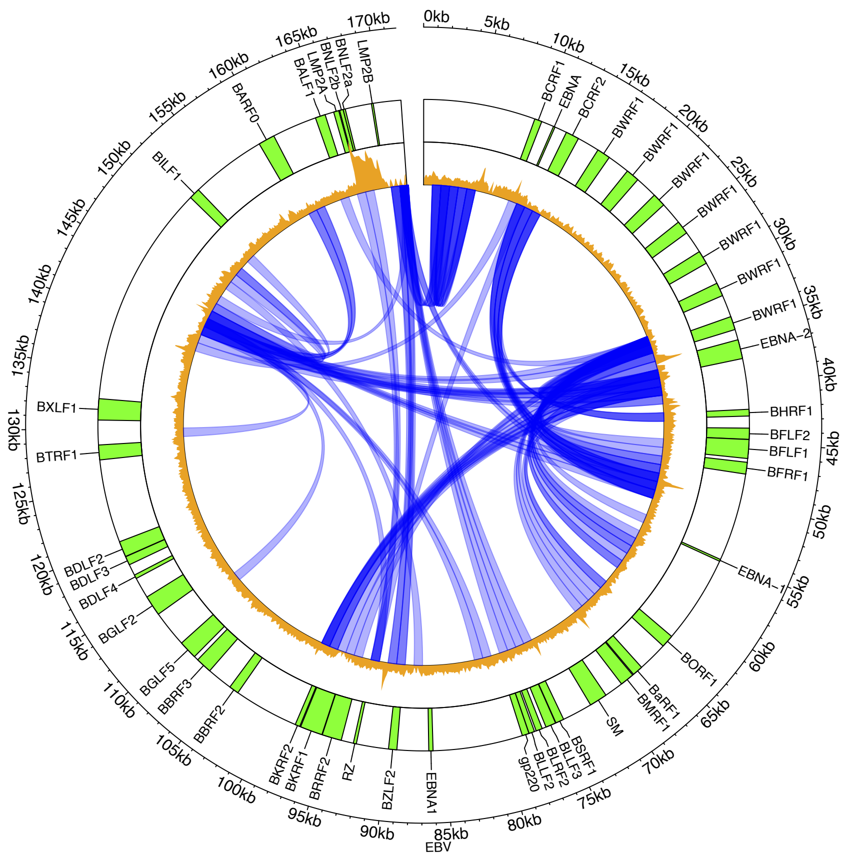


**B)** **Olaparib**

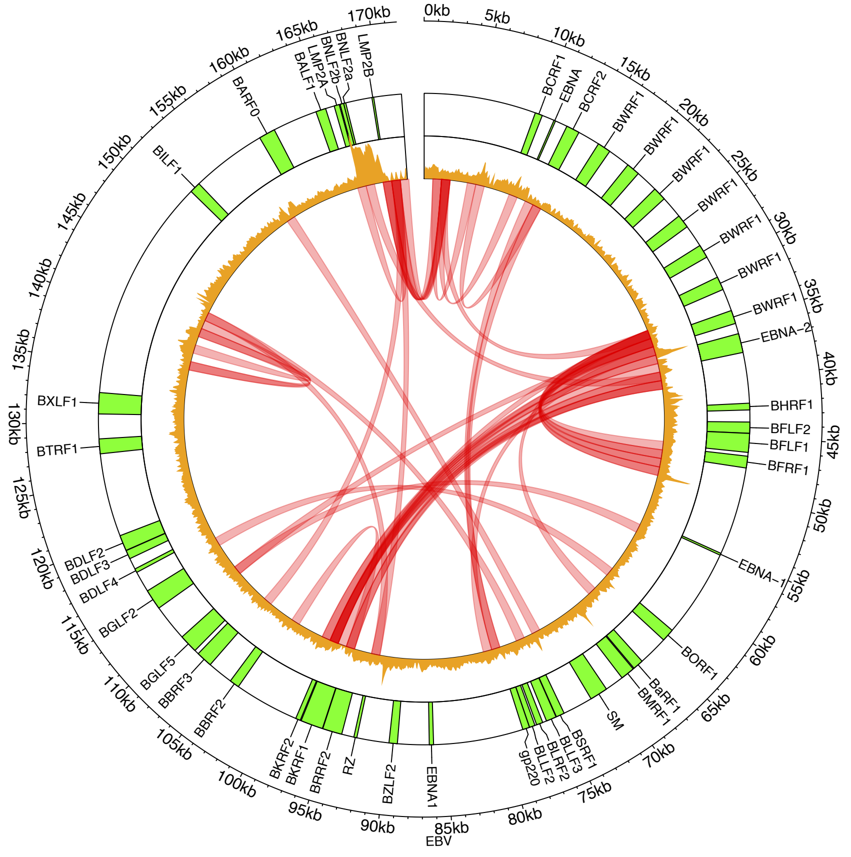

**Supplementary figure 1. CTCF binding profiles aligned to DNA-DNA contact maps.** Circos graphs of all DNA-DNA contacts with significance of < .05 within the type III latency EBV genome (Mutu-LCL) derived from HiC matrices. A) circos graph represents the control (untreated genome, blue) and B) represents the type I genome with 2.5 uM olaparib treatment (red). Darkness of arcs correspond to strength of DNA-DNA contact. CTCF ChIP-seq profile (orange) surrounds the circos graph in untreated (top) and olaparib treated (bottom) Mutu-LCL EBV genome.

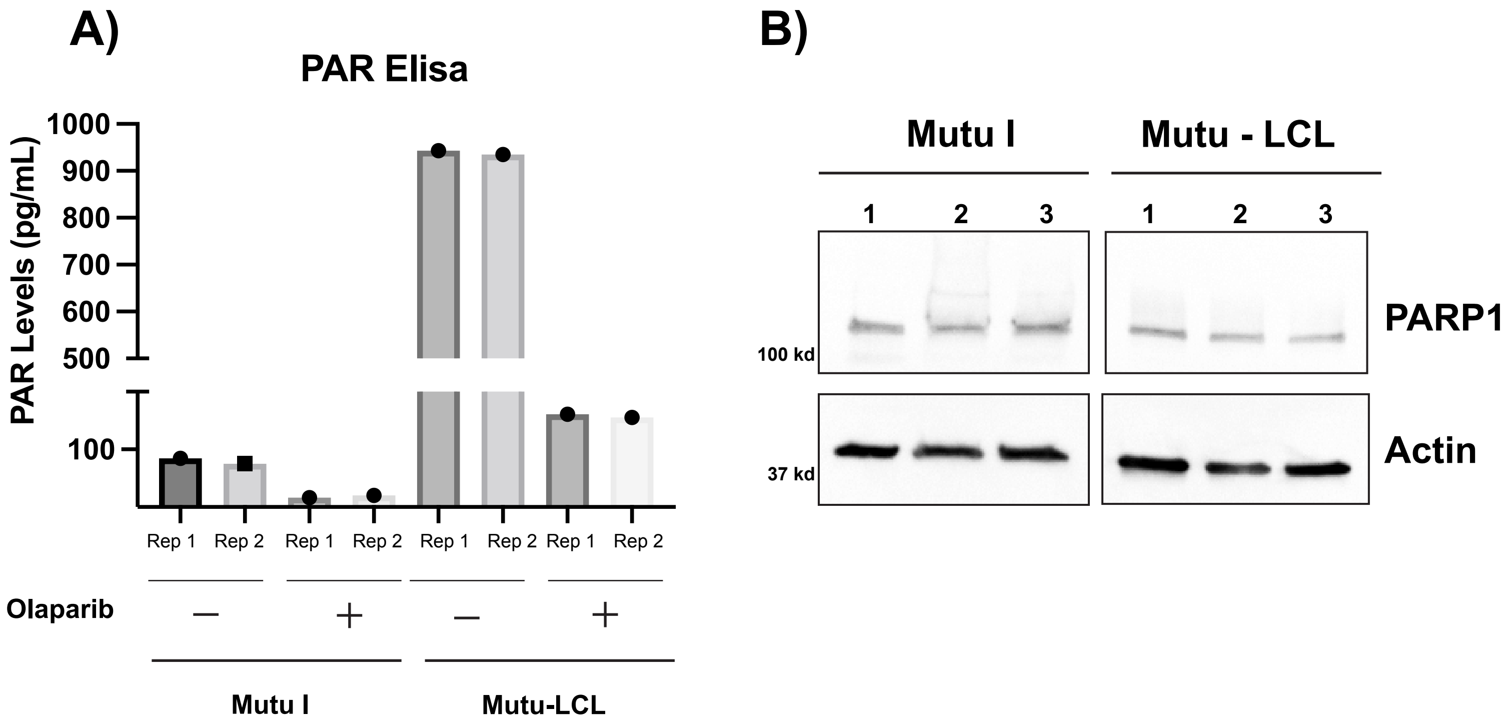
A)
PAR Elisa
PAR Levels (pg/mL)
1000
900
800
700
600
500
100
Rep 1
Rep 2
Rep 1
Rep 2
Rep 1
Rep 2
Rep 1
Rep 2
Olaparib
–
+
–
+
Mutu I
Mutu-LCL
B)
Mutu I
Mutu - LCL
1
2
3
1
2
3
PARP1
100 kd
Actin
37 kd

**Supplemental figure 2. PAR ELISA. A)** Biological duplicates of type I latency Mutu I or type III EBV latency LCL were collected before or after PARP inhibition (2.5uM olaparib for 72 hours). N=2, Mean. Source data are provided as a Source Data file. B) Three biological replicates of Mutu and Mutu-LCL were collected and immunoblotted for PARP1 protein levels as well as actin control. Source data are provided as a Source Data file.

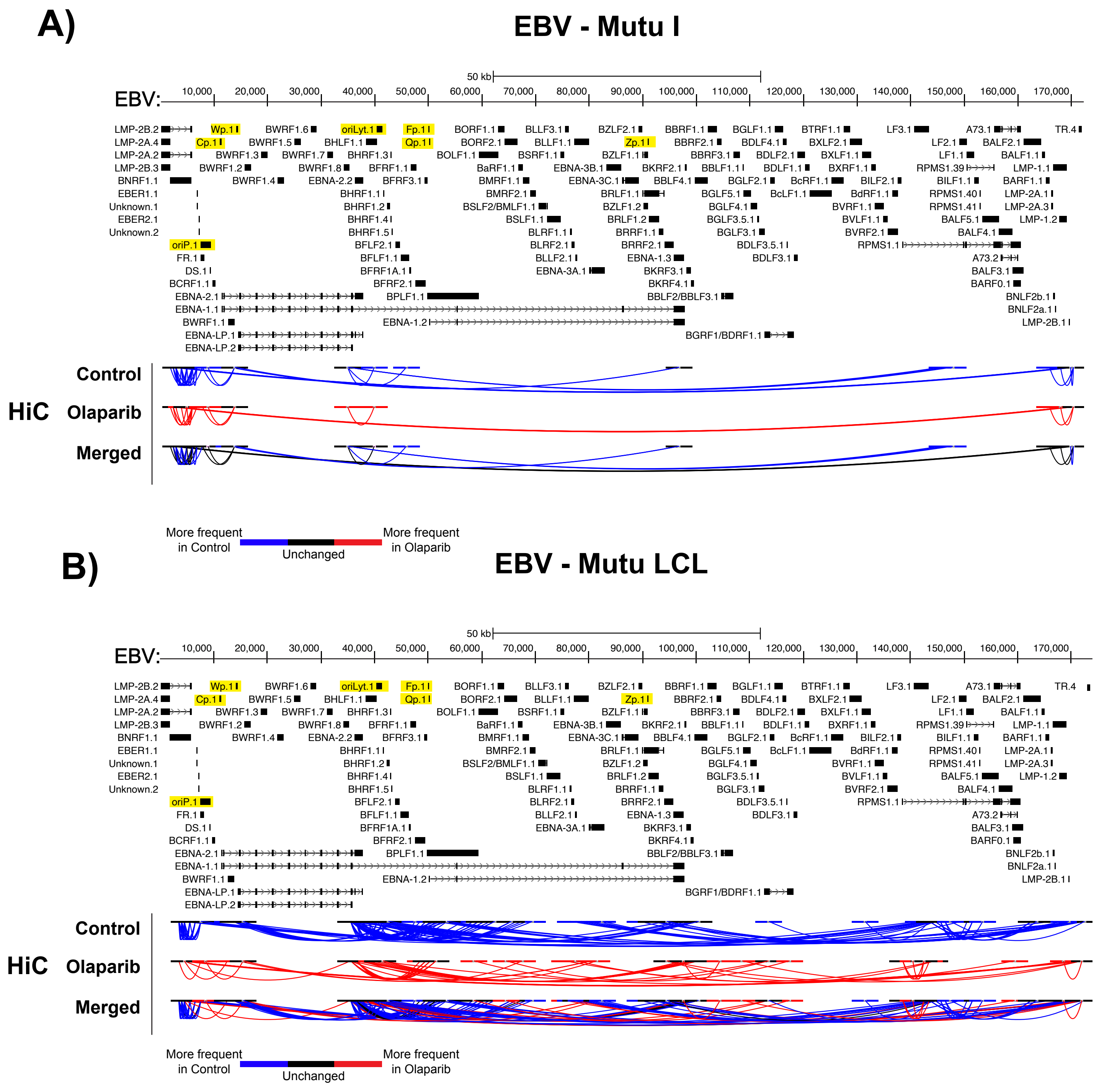
A)
EBV - Mutu I
B)
EBV - Mutu LCL
50 kb
EBV:
HiC
Control
Olaparib
Merged
More frequent in Control
Unchanged
More frequent in Olaparib

**Supplemental figure 3. Linearized depiction of chromatin loops across EBV genomes**. A) Linearized type I EBV genome (Mutu I) with arcs connecting DNA-DNA contacts derived from HiC matrices. Blue arcs represent chromatin loops that are more frequent in control genome, red arcs represent chromatin loops that are more frequently observed in the olaparib treated genome, and black arcs are loops that are unchanged between control and treatment. B) Linearized type III EBV genome (LCL) as described in A.

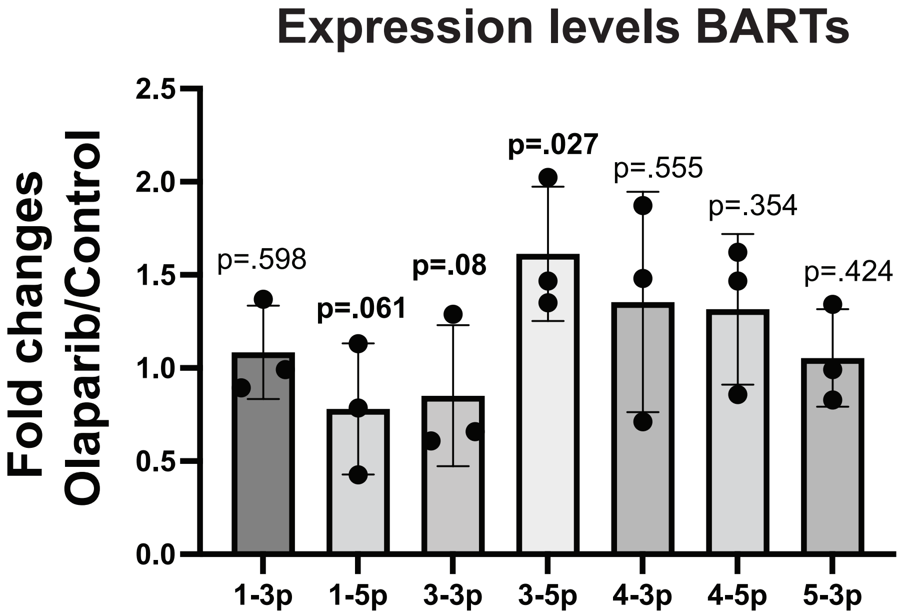
Expression levels BARTs
Fold changes Olaparib/Control
2.5
2.0
1.5
1.0
0.5
0.0
p=.598
p=.061
p=.08
p=.027
p=.555
p=.354
p=.424
1-3p
1-5p
3-3p
3-5p
4-3p
4-5p
5-3p

**Supplemental figure 4. MicroRNA expression in LCL.** RT-qPCR validation of seven randomly selected BART microRNAs, before and after PARP inhibition in LCL. Bar graph represents the average expression of three biological duplicates per treatment, each normalized to U6 snRNA, respectively. Data displayed as fold change of olaparib treated LCL over control (N=3, Mean ± SD). Treatment and control groups were compared by paired student's T-test assuming equal variance; ; (two-tailed). Source data are provided as a Source Data file

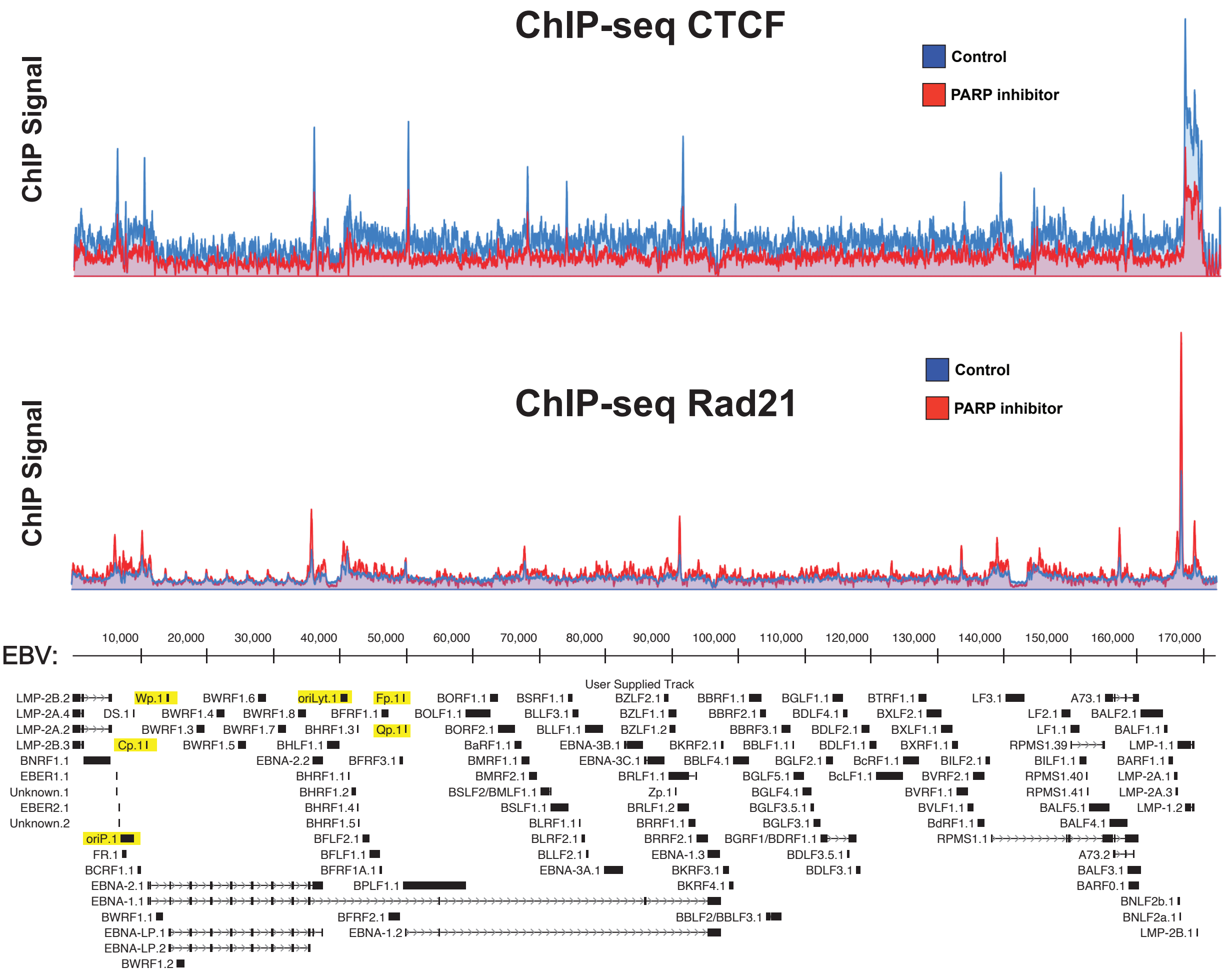
ChIP-seq CTCF
ChIP Signal
Control
PARP inhibitor
ChIP-seq Rad21
ChIP Signal
Control
PARP inhibitor
EBV:
10,000
20,000
30,000
40,000
50,000
60,000
70,000
80,000
90,000
100,000
110,000
120,000
130,000
140,000
150,000
160,000
170,000
User Supplied Track
LMP-2B.2
LMP-2A.4
LMP-2A.2
LMP-2B.3
BNRF1.1
EBER1.1
Unknown.1
EBER2.1
Unknown.2
oriP.1
FR.1
BCRF1.1
EBNA-2.1
EBNA-1.1
BWRF1.1
EBNA-LP.1
EBNA-LP.2
BWRF1.2
Wp.1
DS.1
Cp.1
BWRF1.6
BWRF1.4
BWRF1.3
BWRF1.5
BWRF1.8
BWRF1.7
oriLyt.1
BHRF1.3
BHLF1.1
EBNA-2.2
BHRF1.1
BHRF1.2
BHRF1.4
BHRF1.5
BFLF2.1
BFLF1.1
BFRF1A.1
BPLF1.1
BFRF2.1
EBNA-1.2
Fp.1
BFRF1.1
Qp.1
BFRF3.1
BORF1.1
BOLF1.1
BORF2.1
BaRF1.1
BMRF1.1
BMRF2.1
BSLF2/BMLF1.1
BSLF1.1
BSRF1.1
BLLF3.1
BLLF1.1
EBNA-3B.1
EBNA-3C.1
BLRF1.1
BLRF2.1
BLLF2.1
EBNA-3A.1
BZLF2.1
BZLF1.1
BZLF1.2
BKRF2.1
BRLF1.1
Zp.1
BRLF1.2
BRRF1.1
BRRF2.1
EBNA-1.3
BKRF3.1
BKRF4.1
BBLF2/BBLF3.1
BBRF1.1
BBRF2.1
BBRF3.1
BBLF1.1
BBLF4.1
BGLF5.1
BGLF4.1
BGLF3.5.1
BGLF3.1
BGRF1/BDRF1.1
BDLF3.5.1
BDLF3.1
BGLF1.1
BDLF4.1
BDLF2.1
BDLF1.1
BGLF2.1
BcLF1.1
BTRF1.1
BXLF2.1
BXLF1.1
BXRF1.1
BcRF1.1
BILF2.1
BVRF2.1
BVRF1.1
BVLF1.1
BdRF1.1
RPMS1.1
LF3.1
LF2.1
LF1.1
RPMS1.39
BILF1.1
RPMS1.40
RPMS1.41
BALF5.1
BALF4.1
A73.1
BALF2.1
BALF1.1
LMP-1.1
BARF1.1
LMP-2A.1
LMP-2A.3
LMP-1.2
A73.2
BALF3.1
BARF0.1
BNLF2b.1
BNLF2a.1
LMP-2B.1

**Supplemental figure 5. Mutu-LCL CTCF and RAD21 binding profiles overlaid with and without PARP inhibition.** Read counts from respective ChIP-seq assays were first normalized to the number of reads from input chromatin collected before antibody immunoprecipitation. Normalized reads from CTCF or RAD21 ChIP-seq, before (blue) and after PARP inhibition (red), are aligned together along the EBV genome, viewed in equal scale.

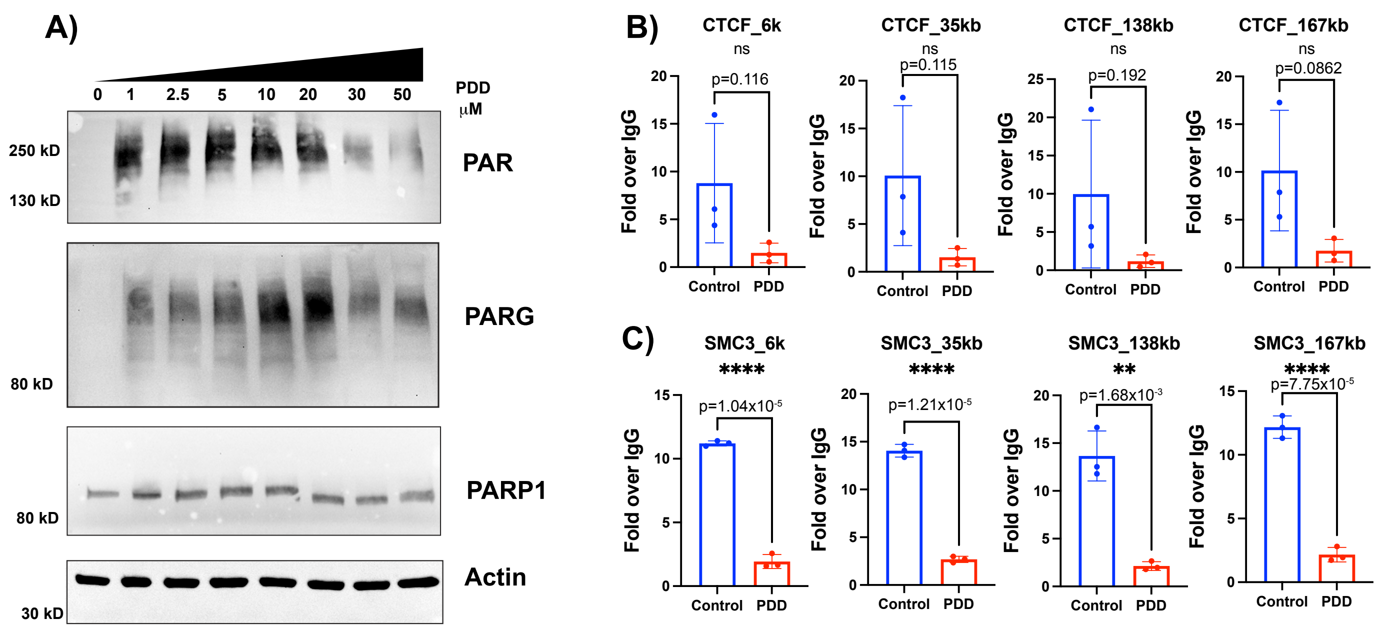

A)
PDD μM
0 1 2.5 5 10 20 30 50
250 kD
130 kD
PAR
PARG
80 kD
PARP1
80 kD
Actin
30 kD
B)
CTCF_6k
ns
p=0.116
CTCF_35kb
ns
p=0.115
CTCF_138kb
ns
p=0.192
CTCF_167kb
ns
p=0.0862
Fold over IgG
Control
PDD
C)
SMC3_6k
****
p=1.04x10-5
SMC3_35kb
****
p=1.21x10-5
SMC3_138kb
**
p=1.68x10-3
SMC3_167kb
****
p=7.75x10-5
Fold over IgG
Control
PDD

**Supplemental figure 6**. CTCF and cohesin ChIP-qPCR with PARG inhibition. A) Immunoblot of poly(ADP-ribose) (PAR), PARG, PARP1, and actin control in Mutu-LCL at increasing concentration of the PARG inhibitor PDD 00017273. Displayed data is representative of three independent assays. Source data are provided as a Source Data file. B-C) ChIP-qPCR of CTCF or cohesin component SMC3 at previously assessed CTCF/cohesin colocalization sites in Mutu-LCL before and after treatment with 2.5 mM of the PARG inhibitor PDD 00017273. Data presented is an average of three independent assays (N=3, Mean $\pm$ SD). Treatment and control groups were compared by paired student's T-test assuming equal variance; ; (two-tailed). (*=p ≤ .05, **=p ≤ .01, ***=p ≤ .001, ****p ≤ .0001). Source data are provided as a Source Data file.

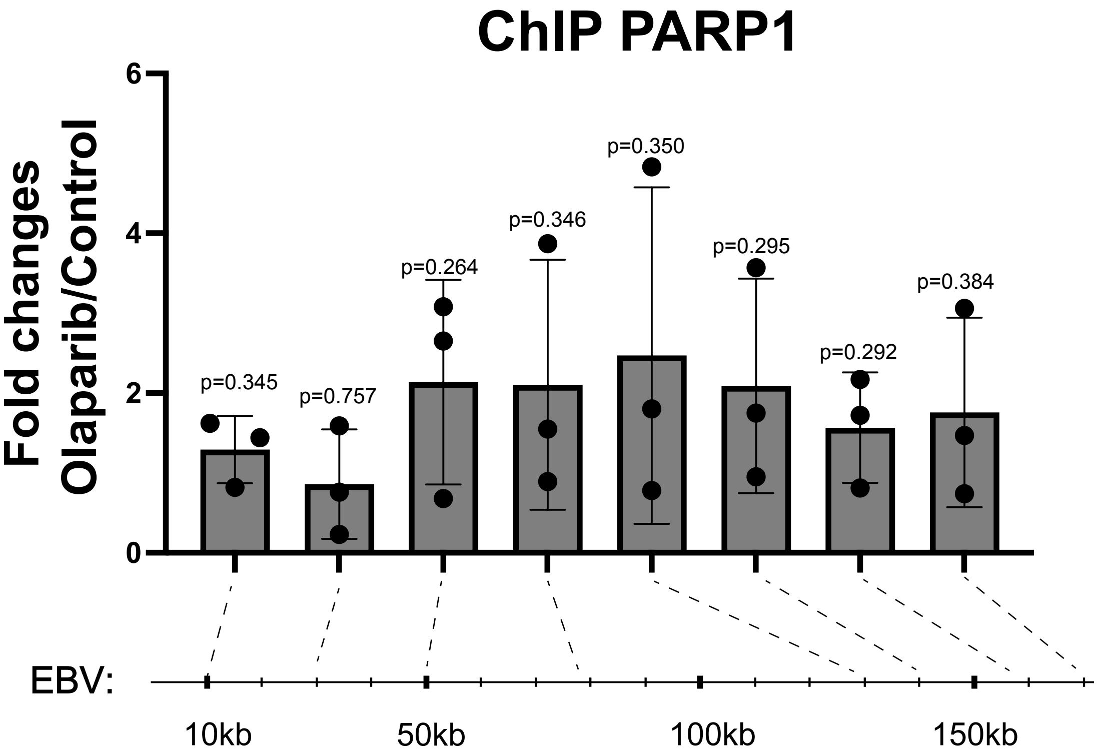
ChIP PARP1
Fold changes
Olaparib/Control
6
4
2
0
p=0.345
p=0.757
p=0.264
p=0.346
p=0.350
p=0.295
p=0.292
p=0.384
EBV:
10kb
50kb
100kb
150kb

**Supplemental figure 7. PARP1 chromatin binding in Mutu-LCL.** ChIP-qPCR of PARP1 binding at previously assessed cohesin/CTCF colocalization sites in Mutu-LCL, as well as four additional PARP1 binding sites along the EBV genome, with and without 2.5uM olaparib treatment. ChIP data was normalized to input chromatin. Data presented is an average of three independent experiments, displayed as fold change of treatment over control. Groups were compared by paired student's T-test assuming equal variance; ; (two-tailed). Source data are provided as a Source Data file
