# Supplementary material for: The three-dimensional structure of Epstein-Barr virus genome varies by latency type and is regulated by PARP1 enzymatic activity
Source: Nat Commun. 2022 Jan 17;13:187. doi: 10.1038/s41467-021-27894-1 (PMC8764100; doi:10.1038/s41467-021-27894-1)
Supplement: Supplementary file 2 — Description of additional Supplementary File [file 41467_2021_27894_MOESM2_ESM.pdf]

Description of additional supplementary files

Supplementary Data 1: Significant chromatin loops identified by Hi-C assay.

Supplementary Data 2: Raw counts that were used in differential interaction analysis for Hi-C assay.

Supplementary Data 3: DNA sequences of primers used in this manuscript
